# Genome-Wide DNA Methylation Analysis Reveals Epigenetic Pattern of *SH2B1* in Chinese Monozygotic Twins Discordant for Autism Spectrum Disorder

**DOI:** 10.3389/fnins.2019.00712

**Published:** 2019-07-17

**Authors:** Shuang Liang, Zhenzhi Li, Yihan Wang, Xiaodan Li, Xiaolei Yang, Xiaolei Zhan, Yan Huang, Zhaomin Gao, Min Zhang, Caihong Sun, Yan Zhang, Lijie Wu

**Affiliations:** ^1^Department of Child and Adolescent Health, School of Public Health, Harbin Medical University, Harbin, China; ^2^Department of Biochemistry and Molecular & Cellular Biology, Georgetown University Medical Center, Washington, DC, United States; ^3^College of Bioinformatics Science and Technology, Harbin Medical University, Harbin, China

**Keywords:** autism spectrum disorder, monozygotic twins, discordant, DNA methylation, *SH2B1*

## Abstract

Autism spectrum disorder (ASD) is a complex neurodevelopmental disorder. Aberrant DNA methylation has been observed in ASD but the mechanisms remain largely unknown. Here, we employed discordant monozygotic twins to investigate the contribution of DNA methylation to ASD etiology. Genome-wide DNA methylation analysis was performed using samples obtained from five pairs of ASD-discordant monozygotic twins, which revealed a total of 2,397 differentially methylated genes. Further, such gene list was annotated with Kyoto Encyclopedia of Genes and Genomes and demonstrated predominant activation of neurotrophin signaling pathway in ASD-discordant monozygotic twins. The methylation of *SH2B1* gene was further confirmed in the ASD-discordant, ASD-concordant monozygotic twins, and a set of 30 pairs of sporadic case-control by bisulfite-pyrosequencing. The results showed that there was a greater DNA methylation difference in ASD-discordant monozygotic twins than ASD-concordant monozygotic twins. Further, verification of the Chr.16:28856743 of *SH2B1* showed significant differences in DNA methylation between case and control. These results suggest abnormal methylation of *SH2B1* is associated with ASD etiology. Our data suggest that it might be worthwhile to further explore the functions of *SH2B1* and related genes of neurotrophin signaling pathway in ASD.

## Introduction

Autism spectrum disorder (ASD) is a group of serious neurodevelopmental disorders defined in an individual by deficits in social interaction and communication, accompanied by restricted interests and stereotypical repetitive behaviors. ASD affects 1–2% of children and confers severe lifelong disability, thus ASD has become a growing clinical and public health problem worldwide (Kim et al., 2011; Elsabbagh et al., 2012; Hill et al., 2015). Traditionally, the etiology for ASD mainly attributed to different genetic variants such as *de novo* mutations, copy number variations (CNV), and single-nucleotide polymorphisms (SNP) (State and Levitt, 2011; Neale et al., 2012). Although focuses on research efforts have grown in intensity during the past decade and due to the highly clinical and etiological heterogeneous nature of ASD, no findings of biological or clinical markers have been definitively identified. This shows that additional epigenetic or environmental factors are an underlying cause for the susceptibility to ASD. A more recent twin study further suggested that an environmental contribution might be a potentially substantial role for ASD etiology (Hallmayer et al., 2011; Schendel et al., 2014). A large survey of over 14,000 children with ASD in Sweden revealed that heritable factors were found to contribute to about half of ASD risk and the other half were contributed from undetected genetic factors, environmental effects and/or stochastic effects.

The epigenetic mechanisms allow stable regulation of gene expression without altering the DNA sequence that is optimally positioned between the genome and the environment (Vogel Ciernia and LaSalle, 2016). Environmentally driven changes have also propelled the development of neuropsychiatric diseases via the alteration of epigenetic profiles, including classic ASD (Schanen, 2006; Grafodatskaya et al., 2010) and symptomatic ASD such as Rett syndrome and Fragile X syndrome. However, environmental modulation of epigenetic states is poorly understood. Since, monozygotic twins have a DNA sequence in common, the study of discordant monozygotic (MZ) twins provides an ideal model to investigate the value of epigenetic factors in the field of disease etiology. DNA methylation, as the most widely investigated process in epigenetic mechanisms, was considered as a key element that mediates reversible changes in gene expression regardless of DNA sequence variation (Henikoff and Matzke, 1997). Recent advances in high-throughput genomic technology expand our knowledge of DNA methylation. As a result, uncovering this complex relationship will be important for understanding the mechanism of DNA methylation in ASD. Lately, two studies have dissected several ASD-associated differentially methylated loci (Nguyen et al., 2010; Wong et al., 2014), and further support that the peripherally derived DNA can be used to identify disease-associated epigenetic changes. Nonetheless, to date, no study has been reported for the methylomic profile in the samples of Chinese ASD-discordant MZ twins, and it is necessary to investigate the significance of epigenetic mechanism in the Chinese population.

This study was designed to identify the genome-wide DNA methylation patterns in Chinese MZ twins discordant for ASD, and further provide the evidence for ASD associated epigenetic alterations.

## Materials and Methods

### Samples Recruitment

All the participants were recruited from the Children Development and Behavior Research Center (CDBRC), Harbin Medical University, Heilongjiang Province, China. Altogether, five pairs of ASD-discordant MZ twins, four pairs of ASD-concordant MZ twins and 30 pairs of sporadic patients with aged-, sex-matched controls were invited to participate. More than two experienced psychiatrists independently issued ASD diagnoses according to the criteria of international Diagnostic and Statistical Manual of Mental Disorders, 5th Ed (DSM-5). All probands were also administered by the Autism Diagnostic Observation Schedule-generic (ADOS) (Lord et al., 2000), and were all found to meet the criteria for autism. The ADOS, a semi-structured observational instrument, is made up for four modules to assess social and communicative abilities in individuals suspected of having ASD, in which two different cut-points that depend on symptom severity result in a diagnosis of (i) ASD (milder variant) or (ii) autism (more severe variant). Cases with Rett syndrome, tuberous sclerosis, fragile-X syndrome, and any other neurological conditions suspected to be associated with autism, were excluded by clinical examination and molecular genetic tests of the *FMR1* gene (Khaniani et al., 2008). The standard biosecurity and institutional safety procedures for the blood collection have been carried out. This study was performed in accordance with the recommendations of Institutional Research Board of Harbin Medical University (HMUIRB2012006). The protocol was approved by the Institutional Research Board of Harbin Medical University. Safety and fairness principle has been fully considered in the study plans. All subjects gave written informed consent in accordance with the Declaration of Helsinki and the content of the research have no harm or risk.

### DNA Extraction and Bisulfite Conversion

Genomic DNA was extracted from whole blood using the QIAamp DNA Mini Kit (Qiagen, Darmstadt, German), and assessed for integrity, quantity, purity and concentration by electrophoresis in a 1% agarose gel and Nanodrop2000 spectrophotometer. Bisulfite conversion of 500 ng of genomic DNA was performed using EZ 96-DNA methylation kit (Zymo Research, Irvine, CA, United States) consistent of manufacturer’s standard protocol. The bisulfite conversion reaction was conducted in duplicate to minimize potential bias caused by variable conversion efficiency, and pooled bisulfite-treated DNA was used for subsequent array experiments.

### Infinium Human-Methylation 450BeadChip (450K) Array

Three pairs of ASD-discordant MZ twins for Genome-wide DNA methylation were measured via the Illumina Infinium Human Methylation 450BeadChip assay (Illumina, San Diego, CA, United States), which interrogated the DNA methylation profile of more than 485,000 methylation sites per sample at single-nucleotide resolution. Arrays were scanned by HiScan 2000 (Illumina). Illumina Genome Studio software (Illumina) was used to extract signal intensities for each probe and to perform initial control quality checks. There were no failed samples which needed to be excluded by examined 450K array control probes to assess bisulfite conversion, extension, hybridization, staining, specificity, negative control and others; The ChAMP pipeline was used to normalize and batch correct methylation array data (Johnson et al., 2007; Teschendorff et al., 2013; Morris et al., 2014). Probes that ambiguously mapped or had a high detection *p-*Value (>0.01), low bead count (<3 beads), and a low success rate (missing in >95% of the samples) were set to missing. Probes with SNPs (MAF ≥ 0.05 in 1000 Genomes Project) at CpG sites were excluded to avoid SNP (single-nucleotide polymorphism) effects on methylation measurement. To investigate if blood cell-composition was similar between the MZ twins, cell type counts (“CD8T,” “CD4T,” “NK,” “Bcell,” “Mono,” and “Gran”) were estimated using the method of Houseman et al. (2012). The difference in methylation level for each pair of twins was calculated by a paired *t*-test, and it was found that there was no significant difference in blood cell components between them (*p* > 0.05) (see Supplementary Table 5).

### Reduced Representation Bisulfite Sequencing (RRBS)

Two pairs of ASD-discordant MZ twins for Genome-wide DNA methylation were assessed using RRBS (Gu et al., 2011), which contained over 1 million CpG sites. RRBS was an innovative method that specifically enriched genomic regions, which has a high density of potential methylation sites and enabled investigation of DNA methylation at single-nucleotide resolution, and coupled bisulfite conversion and next generation sequencing. 5 μg of genomic DNA was digested overnight with 4 μl MspI (New England Biolabs, Ipswich, MA, United States) to ensure complete digestion and was purified by phenol-chloroform extraction. After end repair and addition of 3′A overhangs, methylated-adaptors were ligated according to the manufacturer’s protocol and purified with AMPure beads (Agencourt Bioscience, Beverly, MA, United States). Two ranges of 150–175 and 175–225 bp adapter-ligated fractions were excised from a 2% agarose gel, respectively. Bisulfite conversion was conducted using the reagents and protocol of EpiTect Bisulfite Kit (Qiagen, Darmstadt, Germany). Subsequently, the bisulfite-converted DNA was amplified by PCR with PfuTurbo Cx Hotstart DNA polymerase to build the PCR-amplified RRBS libraries, which were analyzed by Illumina HiSeq2500 (Illumina). We then used whole genome bisulfite sequence mapping program (BSMAP version 2.74) to map the raw sequencing reads to GRCh37/hg19 reference genome and obtain methylation values of CpG sites. The numbers of unconverted and converted cytosine (C) reads covering the loci for each sample were extracted using python scripts methyratio.py in the BSMAP package.

### Methylation Microarray and RRBS Data Processing

Our analysis were performed by R scripts^1^ and Perl scripts^2^. Some of the R packages were coded by us and can be applied with request. Overlap of 450K and RRBS data was generated by findOverlap function in GenomicRanges Bioconductor package (Lawrence et al., 2013). MethylKit Bioconductor package was used for methylation correlation and clustering analysis (Akalin et al., 2012; Lawrence et al., 2013). The relative methylation level of each interrogated CpG site was calculated as the ratio of the normalized signal from the methylated probe to the sum of the normalized signals of the methylated and unmethylated probes. A DNA methylation value, described as “β-value” for each CpG site, ranges from 0 (unmethylated) to 1 (fully methylated). β-values with detection *p*-Value > 0.01 were considered to fall below the minimum intensity and threshold and were consequently removed.

### Differentially Methylated CpG Sites Screening

An analytical approach taking advantage of the discordant MZ twin design was applied in order to maximize our chances of identifying real within-twin differences in DNA methylation. Our analysis consisted of two separate tests. The first was a fold-change with the purpose of assessing the significance of DNA methylation differences between the affected and unaffected member in each twin pair, and the second was the one of assessing the size of methylation differences: a delta-β (Δβ) value was calculated representing the mean difference in methylation between the affected and unaffected. We set parameters (fold change ≥ 2 or ≤ 0.5 or |Δβ| ≥ 0.1) as cutoff, because we wanted to be slightly more inclusive and were willing to test a larger number of differentially methylated CpG sites in our downstream replication analyses. The CpG sites which met one of criterions were included, and the two differential loci lists were mapped to UniProt–SwissProt to obtain gene list. In DNA methylation analysis, genes are annotated based on the relative promoter regions, since the primary method for DNA methylation to regulate transcription is through inhibiting transcription binding sites to methylated cytosine at promoter sites upstream of start codon. Further, these genes were taken intersection across all twin pairs. GRCh37 (UCSC hg19, Feb/2009) was used as human genome reference sequence.

### Functional Enrichment Analysis of Differentially Methylated Genes

The enrichment analysis for biological processes and gene networks relevant to the pathogenesis of ASD, based on the differentially methylated genes, was conducted using DAVID (version 6.7) online software^3^. To understand the functions and utilization of biological systems, the intersected list of differential methylation genes (Entrez gene ID annotated) was uploaded into the Kyoto Encyclopedia of Genes and Genomes (KEGG) pathway (Kanehisa et al., 2017), showing the molecular interactions and reaction networks. Hypergeometric model was used to calculate *p* value and to determine if the number of genes is greater than expected. *P*-value was subsequently adjusted for multiple testing.

### Methylation Pyrosequencing

Pyrosequencing was employed to further validate differences in DNA methylation identified by 450K array and RRBS. Briefly, 500 ng DNA from each individual was treated with sodium bisulfite, which used the EZ96-DNA methylation kit in light of the manufacturer’s recommendation, and amplified by a bisulfite polymerase chain reaction. Quantitative DNA methylation analysis of each CpG was conducted using PSQ96 Pyrosequencer (Qiagen). Primers were designed with an online program MethPrimer^4^ for Primer F (5′-TTGTATGATTAGGGTAGTTTGTGTGG-3′), primer R (5′-ATTCTTTTCCTCCTTTAATTCTAACT-3′) and sequencing Primer (5′-GTATTGATGGTGATATT-3′) to amplify an amplicon with 174 bp, which corresponded to the region detected by the 450K array and RRBS. The sequence of primers was blasted against a gene bank in NCBI.

## Results

### Genome-Wide DNA Methylation Profiling Analysis

**FIGURE 1:**
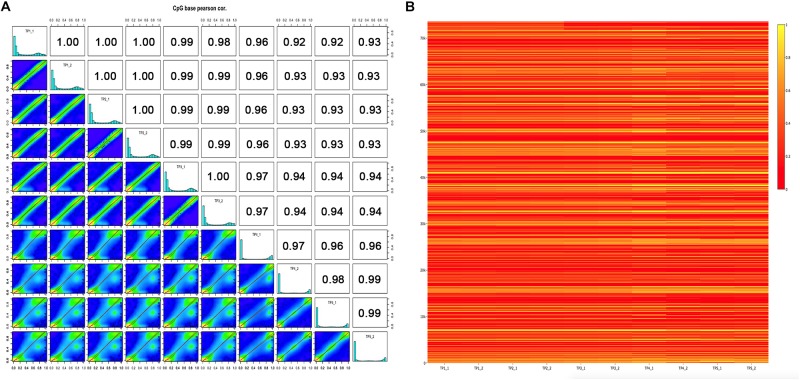
DNA methylation levels of five ASD-discordant monozygotic twins obtained in 450K array and RRBS. **(A)** Scatter plots for sample pairs. Scatter plots of % methylation values for each pair in five ASD-discordant monozygotic twins. Numbers on upper right corner denote pair-wise Pearson’s correlation scores. The histograms on the diagonal are % methylation histograms per cytosine for each sample. Most of the bases have either high or low methylation. **(B)** Heat map of methylation CpG sites of five ASD-discordant monozygotic twins. Scaled methylation values are color-coded according to the legend on the right.

To determine the genome-wide DNA methylation signal in five pairs of MZ discordant autism, we applied two methods, Infinium 450K array and RRBS from individuals with an ADOS-confirmed diagnosis of autism and their control sibling. Demographic data of the samples involved in this study are presented in Supplementary Table 1. Metrics from array and RRBS passed standard quality control in our analyses. In each sample, % methylation had a bimodal distribution, which indicated that the majority of bases had either high or low methylation. Pairwise Pearson correlation coefficients were calculated among the ten samples within five ASD-discordant MZ twins, and to produce a correlation matrix, which easily compare correlation coefficients between pairs of samples. As expected, a high correlation was observed in genome-wide DNA methylation within each MZ twin (ranged between 0.97 and 1.00), indicating that ASD is not co-related with systemic changes in epigenetic programming (Figure 1A). In order to further verify the similarities observed in methylation status within five ASD-discordant MZ twins, an integrated heat map was generated, which displayed no significant differences in global DNA methylation profile (Figure 1B).

### Identification of Differentially Methylated CpG Sites

Although there were no significant differences in global DNA methylation levels, DNA methylation at individual CpG sites indicated substantial differential methylated CpGs within ASD-discordant MZ twins pairs, as the phenotypes involved in the comparisons were different. A myriad of sites throughout the genome exhibiting disease-associated differential DNA methylation have been observed within co-twins by combined analysis method (difference and fold change), and these have been further mapped to genes (Table 1). Next, the intersected genes were obtained from two sets of genome-wide methylation data. In the 450K array dataset, there were 2,581 differentially methylated genes intersected in three ASD-discordant MZ pairs and 10,506 differentially methylated genes intersected from RRBS data in two ASD-discordant MZ pairs. Overall, 2,397 intersected genes were identified between the two datasets of five pairs of ASD-discordant MZ twins.

**TABLE 1:** Differential methylated loci and genes identified by genomic-wide methylation platform.

**Sample**	**platform**	**Differential methylated loci**	**Differential methylated genes**
TP1	450K	14,241	4,661
TP2	450K	13,461	4,566
TP3	450K	27,729	6,645
TP4	RRBS	510,159	11,108
TP5	RRBS	409,381	10,812

*TP, twin pair; cutoff parameters (fold change ≥ 2 or ≤ 0.5 or |Δβ| ≥ 0.1).*

### Functional Enrichment Analysis of Differentially Methylated Genes

**FIGURE 2:**
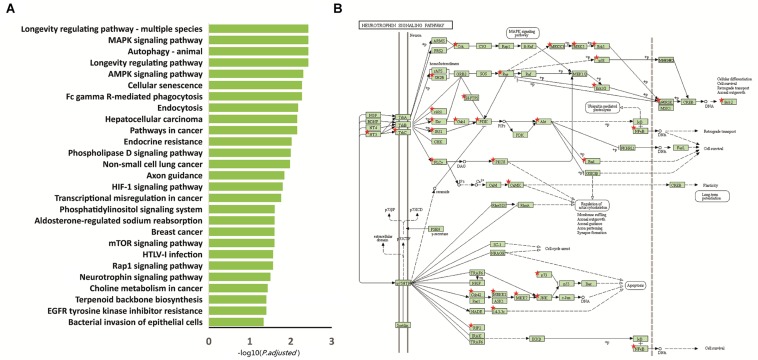
Functional enrichment analysis of the 2,397 intersected differential genes. **(A)** Relevant pathway enriched of 2,397 intersected differential genes in KEGG pathway. **(B)** Intersected differential genes enriched in the neurotrophin signaling pathway. Red star: different methylation genes.

We performed functional enrichment analysis based on the Kyoto Encyclopedia of Genes and Genomes (KEGG) for the 2,397 differentially methylated intersected genes (see Supplementary Table 2) to examine whether common networks or pathways were over-represented in the list of genes associated with the differentially methylated CpG sites for autism. The results showed that 27 pathways were significantly enriched (*p.adjust* < 0.05) (Figure 2A and Table 2). Of these pathways, the neurotrophin signaling pathway (KEGG: hsa04722) was selected for further study, because it is involved in many processes in the central nervous system and plays a critical role in the neurogenesis, differentiation and synaptic plasticity (Pardo and Eberhart, 2007; Segura et al., 2015). Indeed, accumulating evidence suggests that neurotrophic factors might play a crucial role in the ASD pathogenesis and represent a group of candidate genes for ASD etiology (Nickl-Jockschat and Michel, 2011). Given these properties, the neurotrophin signal pathway is promising candidates for influencing ASD pathophysiology. Thirty-five genes were enriched in this pathway, including *NFKB1*, *IRAK4*, *CDC42*, *KRAS*, *MAP3K3*, *MAP3K1*, *BCL2*, *GAB1*, *CAMK2D*, *PIK3CA*, *CAMK2B*, ***SH2B1***, *SHC3*, *PIK3R3*, *MAP2K7*, *AKT3*, *PIK3R1*, *MAP2K5*, *NTF3*, *BAD*, *MAPK10*, *IRS1*, *TP73*, *PTPN11*, *NTRK3*, *MAPK1*, *CRKL*, *MAPK12*, *PLCG1*, *RPS6KA1*, *RPS6KA2*, *MAPK14*, *MAPK7*, *ABL1*, and *PDPK1* (Figure 2B).

**TABLE 2:** The significant pathways enriched in KEGG.

**Pathway ID**	**Pathway name**	***p.adjust***
KEGG: hsa04211	Longevity regulating pathway	0.004
KEGG: hsa04140	Autophagy – animal	0.004
KEGG: hsa04010	MAPK signaling pathway	0.004
KEGG: hsa04213	Longevity regulating pathway – multiple species	0.004
KEGG: hsa04152	AMPK signaling pathway	0.005
KEGG: hsa04666	Fc gamma R-mediated phagocytosis	0.005
KEGG: hsa04218	Cellular senescence	0.005
KEGG: hsa04144	Endocytosis	0.007
KEGG: hsa05200	Pathways in cancer	0.007
KEGG: hsa05225	Hepatocellular carcinoma	0.007
KEGG: hsa01522	Endocrine resistance	0.010
KEGG: hsa04072	Phospholipase D signaling pathway	0.010
KEGG: hsa05223	Non-small cell lung cancer	0.010
KEGG: hsa04360	Axon guidance	0.014
KEGG: hsa04066	HIF-1 signaling pathway	0.016
KEGG: hsa05202	Transcriptional misregulation in cancer	0.017
KEGG: hsa04070	Phosphatidylinositol signaling system	0.025
KEGG: hsa04960	Aldosterone-regulated sodium reabsorption	0.025
KEGG: hsa04150	mTOR signaling pathway	0.025
KEGG: hsa05224	Breast cancer	0.025
KEGG: hsa05166	HTLV-I infection	0.027
KEGG: hsa04015	Rap1 signaling pathway	0.027
KEGG: hsa04722	Neurotrophin signaling pathway	0.031
KEGG: hsa05231	Choline metabolism in cancer	0.036
KEGG: hsa01521	EGFR tyrosine kinase inhibitor resistance	0.039
KEGG: hsa00900	Terpenoid backbone biosynthesis	0.039
KEGG: hsa05100	Bacterial invasion of epithelial cells	0.046

### Methylation Pyrosequencing

Further analyses sought to determine if these 35 candidate genes were highly represented among differentially methylated genes that are relevant to neurological functions and disorders in other samples. We compared these genes that displayed differentially methylated CpGs in neurotrophin signaling pathway to the electronic database (PubMed). An extensive literature review was conducted on these genes that are linked to ASD. Among them, 30 (85.7%) genes had been implicated in genetics studies on ASD (Xu et al., 2012; Supplementary Table 3). Of particular note is *SH2B1*, an interesting gene chosen for further bisulfite-pyrosequencing analysis, owing to the location in 16p11.2, which is one of the most frequent genetic etiologies of ASD. Furthermore, there is a greater incidence of obesity in the ASD population and *SH2B1* is reported to be causative for obesity. Following up on this result, a replication analysis was verified for three methylation sites (Chr.16:28856729, Chr.16:28856735, and Chr.16:28856743) of *SH2B1* in ASD-discordant MZ twins, ASD-concordant MZ twins and a set of 30 pairs sporadic case-control sample.

### Five ASD-Discordant MZ Twins

To obtain high-resolution information about specific CpG sites differentially methylated in autism, further validation of disease-associated DNA methylation (Chr.16:28856729, Chr.16:28856735, and Chr.16:28856743) located in *SH2B1* was verified by bisulfite-pyrosequencing within five ASD-discordant MZ twins. The size of absolute methylation difference (|Δβ| ≥ 0.1) was used as a cutoff. The Chr.16:28856729 was identified in twin pair 2 exhibiting autism, compared to the unaffected sibling (|Δβ| = 0.1199). The Chr.16:28856743 methylation difference was shown to be associated with ASD in both twin pair 2 (|Δβ| = 0.2436) and twin pair 5 (|Δβ| = 0.2266). The Chr.16:28856735 did not reveal any methylation difference in bisulfite-pyrosequencing, which is likely a disagreement between the high throughput approaches and bisulfite-pyrosequencing. While no overall significant difference existed in DNA methylation at the 450K array and RRBS nominated sites were observed across the five ASD-discordant MZ twins, it was striking that 2 out of the 5 (40%) pairs tested showed marked methylation difference and were clear outliers at *SH2B1* (Table 3).

**TABLE 3:** Methylation status identified candidate sites of *SH2B1* in five ASD-discordant MZ twins by pyrosequencing.

**Samples**	**Methylation status**			
	
	**Chr.16:28856729**	**Chr.16:cg28856735**	**Chr.16:cg28856743**	**Mean**
TP1_1	0.6119	0.7808	0.7109	0.7012
TP1_2	0.6174	0.7890	0.7609	0.7224
|Δβ|	0.0055	0.0082	0.0500	0.0212
TP2_1	0.6311	0.8166	0.7564	0.7347
TP2_2	0.7510	0.8455	1.0000	0.8655
|Δβ|	0.1199^*^	0.0289	0.2436^*^	0.1308^*^
TP3_1	0.4491	0.5842	0.5724	0.5352
TP3_2	0.4262	0.6019	0.5835	0.5372
|Δβ|	0.0229	0.0177	0.0111	0.0020
TP4_1	0.4938	0.6558	0.6268	0.5921
TP4_2	0.4953	0.6667	0.6533	0.6051
|Δβ|	0.0015	0.0109	0.0265	0.0130
TP5_1	0.7204	0.8376	1.0000	0.8527
TP5_2	0.6308	0.7849	0.7734	0.7297
|Δβ|	0.0896	0.0527	0.2266^*^	0.1230^*^

TP, twin pair (_1, case and _2, control); |Δβ|, methylation difference; cutoff parameters, |Δβ| ≥ 0.1; ^*^significant.

### Four ASD-Concordant MZ Twins

The methylation status of the identified candidate sites of *SH2B1* were tested by bisulfite-pyrosequencing in four ASD-concordant MZ twins. The results showed that no methylation differences at *SH2B1* were observed in ASD-concordant MZ twins, which showed a consistent methylation profile across ASD-concordant MZ twins. The results further suggest that greater DNA methylation differences occurred in ASD-discordant MZ twins than in ASD-concordant MZ twins (Table 4).

**TABLE 4:** Methylation status identified candidate sites of *SH2B1* in four ASD-concordant MZ twins by pyrosequencing.

**Samples**	**Methylation status**			
	
	**Chr.16:28856729**	**Chr.16:cg28856735**	**Chr.16:cg28856743**	**Mean**
TP6_1	0.6257	0.7862	0.7806	0.7308
TP6_2	0.6449	0.8367	0.7579	0.7465
|Δβ|	0.0192	0.0505	0.0227	0.0157
TP7_1	0.6191	0.7831	0.8024	0.7349
TP7_2	0.6424	0.7955	0.8371	0.7583
|Δβ|	0.0233	0.0124	0.0347	0.0235
TP8_1	0.7892	0.9368	0.9240	0.8833
TP8_2	0.7462	0.8817	0.9476	0.8585
|Δβ|	0.0430	0.0551	0.0236	0.0248
TP9_1	0.4755	0.6610	0.5839	0.5735
TP9_2	0.4944	0.6442	0.5705	0.5697
|Δβ|	0.0189	0.0168	0.0134	0.0038

*TP, twin pair (_1 and _2, both cases); |Δβ|, methylation difference; cutoff parameters, |Δβ| ≥ 0.1.*

### Thirty Pairs of Sporadic Age- and Sex- Match Autism and Controls

**FIGURE 3:**
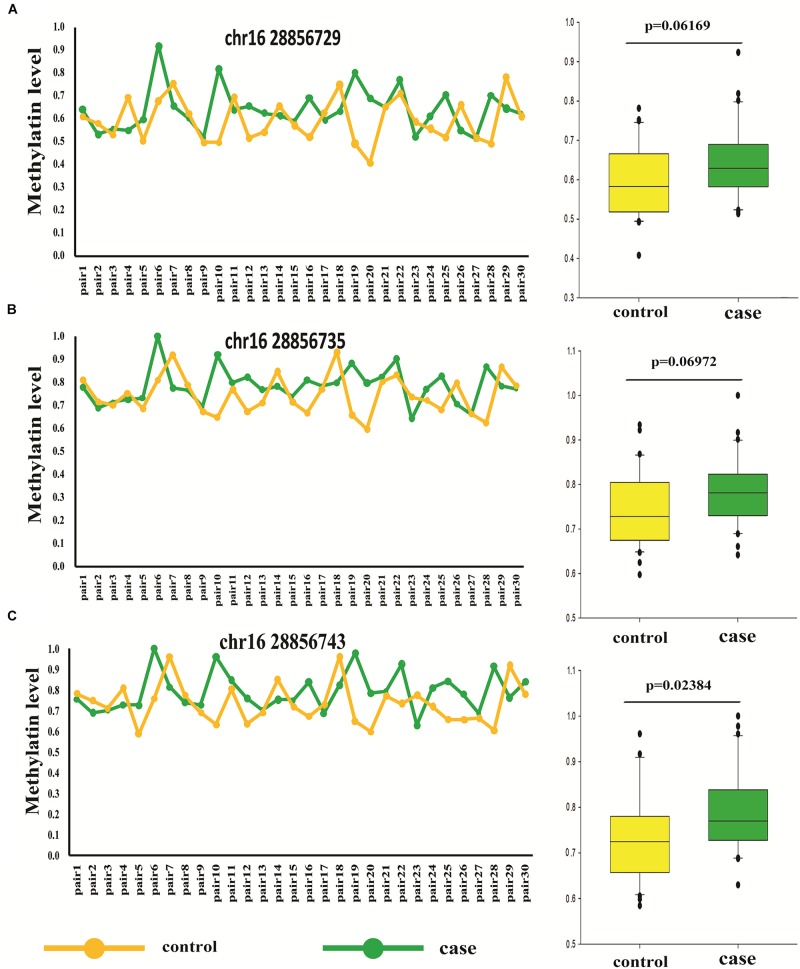
Different methylation loci status of *SH2B1* in autism and controls by pyrosequencing (pair *t*-test). **(A)** Methylation level of Chr.16:28856729 of *SH2B1* in case and control. **(B)** Methylation level of Chr.16:28856735 of *SH2B1* in case and control. **(C)** Methylation level of Chr.16:28856743 of *SH2B1* in case and control. Green, case and yellow, control.

To further confirm the accuracy of high throughput data, bisulfite-pyrosequencing was performed on Chr.16:28856729, Chr.16:28856735, and Chr.16:28856743 located at *SH2B1* in 30 pairs of sporadic ADOS-diagnosis autism, in comparison to the respective age- and sex-matched controls (see Supplementary Table 4). The DNA methylation on Chr.16:28856743 showed was significantly different between autism and unaffected control (paired *t*-test, *p* = 0.035). The mean of these three methylation differential sites also showed statistical significance (paired *t*-test, *p* = 0.023), which illustrated the correlation between autism and methylation at this region (Figure 3 and Supplementary Table 4).

## Discussion

In summary, our study showed the novel finding of the epigenetic effect on ASD risk, while genetic factors were controlled. Differences of disease-associated DNA methylation were analyzed comprehensively in Chinese MZ twins discordant for ASD, using two genome-wide approaches. No alterations appeared between affected and unaffected twins in global DNA methylation, whereas, considerable disease-associated differences between-twins at specific loci across the genome have been successfully observed. We identified disease-associated DNA methylation genes which were not previously implicated in ASD. In addition, our data replicated other studies which revealed differential methylated genes implicated in ASD (Nguyen et al., 2010). Pathway analysis of the intersected differential genes highlighted a significant enrichment of epigenetic disruption of neurotrophin signaling pathway, which is relevant to psychiatric disease and neurodevelopment disorders. Specifically, *SH2B1*, one of the candidate genes whose methylation status was disturbed and further confirmed by bisulfite-pyrosequencing. Overall, our data are proved to support the role of DNA methylation differences in ASD etiology.

There are two reasons to choose *SH2B1* for further analysis. First, *SH2B1* is located in 16p11.2, which is the one of the most common chromosomal abnormal regions in autism (Veenstra-Vanderweele et al., 2004). The mounting evidences indicated that microdeletion and microduplication in 16p11.2 had been closely related to a wide range of neurodevelopmental conditions including ASD (Kumar et al., 2008; Weiss et al., 2008). Tabet et al. (2012) reported three children of a family with autism, including two monozygotic twins, carrying different rearrangements on the chromosome 16p11.2. Notably, our preliminary research using Affymetrix CytoScan HD platform had demonstrated CNV in 16p11.2 in Han Chinese individuals with ASD (Gazzellone et al., 2014), which suggested that the epigenetic factors of *SH2B1* located in 16p11.2 was also involved in ASD.

Second, pediatric overweight and obesity are significant public health concerns. Childhood obesity may persist into adolescence or adulthood and have an increased risk of development related metabolic disorders, including impaired glucosetolerance, hyperinsulinemia, dyslipidemia, type 2 diabetes, non-alcoholic fatty liver disease, and cardiovascular disease (Quattrin et al., 2005; Weiss et al., 2009; Reilly and Kelly, 2011; Katzmarzyk et al., 2012). It is noteworthy that there is a greater incidence of obesity in the ASD population as compared to the general population (Curtin et al., 2005, 2010; Cunningham et al., 2014; de Vinck-Baroody et al., 2015; Dreyer Gillette et al., 2015; Hill et al., 2015). Particularly, the region harboring the *SH2B1* gene was reported in early-onset obesity (Bochukova et al., 2010). *SH2B1* also participated in neurodevelopmental and other phenotypes, which could promote neurite outgrowth of PC12 cells, hippocampal and cortical neurons (Chen et al., 2015). Deletions of the 16p11.2 harboring *SH2B1* were pathogenic and were linked to developmental delay in addition to obesity (Bachmann-Gagescu et al., 2010). Maillard et al. (2015) also showed that the 16p11.2 locus regulated brain structures common to autism and obesity. Criado et al. (2017) indicated that identification of factors of obesity risk in children with ASD served as a prerequisite for addressing long-term individual health burdens and societal costs in this population. The data reported here suggested that epigenetic changes of *SH2B1* might confer risk of ASD.

However, there are considerable limitations of the study. First, DNA was extracted from blood rather than the brain tissue. Although we know methylation is the characteristic of tissue-specificity, it is difficult to obtain the brain tissue of ASD. Moreover, the mounting evidences have supported disease-associated methylation loci could be identified from peripheral samples. *BCL2* previously reported in brain tissue (Nguyen et al., 2010) was also identified in peripheral blood from our study. Second, the sample size in each group was small. In our study, there were 9 pairs of ASD MZ twins involved. Although ASD-discordant MZ twins and ASD-concordant MZ twins are relatively rare, further evaluation in larger sample is warranted to confirm our findings. Finally, since methylation in regulating gene expression plays a vital role, it is reasonable that CNV and methylomic variation could mediate disease susceptibility through the alteration of gene dosage (Wong et al., 2014). If gene dosage alteration of *SH2B1* was obtained, we can further confer the risk of ASD. RNA was not available from peripheral blood when collecting samples, so we have no RNA expression data. Therefore, we could not directly evaluate *SH2B1* gene expression. In future study, CNV and RNA expression data should be further added in order to identify discrete *SH2B1* showing methylation differences between autism and controls.

The present study suggest that it may also be worthwhile to further explore how *SH2B1* and related genes on neurotrophin signaling pathway affect ASD and risk of obesity in the ASD population.

## Ethics Statement

This study was carried out in accordance with the recommendations of the Institutional Research Board of the Harbin Medical University (HMUIRB2012006). The protocol was approved by the Institutional Research Board of the Harbin Medical University. Safety and fairness principle has been fully considered in the study plans. All subjects gave written informed consent in accordance with the Declaration of Helsinki and the contents of the research have no harm or risk.

## Author Contributions

SL contributed to the array and molecular biology experiments, carried out the data analysis, and wrote the manuscript. ZL, YW, and MZ carried out the data analysis. XY, XL, and XZ contributed to the array and molecular biology experiments. YH, CS, and ZG contributed to the sample collection and data collection. YZ and LW were responsible for the study design, protocol development, interpretation of data, and revised the manuscript. All authors have approved the final version of the manuscript.

## Conflict of Interest Statement

The authors declare that the research was conducted in the absence of any commercial or financial relationships that could be construed as a potential conflict of interest.

## Footnotes

1 http://www.r-project.org

2 https://www.perl.org/

3 https://david.ncifcrf.gov/

4 https://www.urogene.org/methprimer/
