# Supplementary material for: Genome-Wide DNA Methylation Analysis Reveals Epigenetic Pattern of SH2B1 in Chinese Monozygotic Twins Discordant for Autism Spectrum Disorder
Source: Front Neurosci. 2019 Jul 17;13:712. doi: 10.3389/fnins.2019.00712 (PMC6660254; doi:10.3389/fnins.2019.00712)
Supplement: Supplementary file 1 [file Table_1.DOCX]

**Methylomic Analysis reveals Epigenetic Pattern of *SH2B1* in Chinese Monozygotic Twins Discordant for Autism Spectrum Disorder**

**Supplementary Table 1 Description of clinical details used in the methylation analyses**

| Case no. | Sex | Age | Nation | Diagnosis | Sample type | Methods |
| --- | --- | --- | --- | --- | --- | --- |
| **Case** |  |  |  |  |  |  |
| TP1_1 | Female | 12 | Han | autism | ASD-discordant MZ twins | Illumina 450K,  pyrosequencing |
| TP2_1 | Female | 2 | Han | autism | ASD-discordant MZ twins | Illumina 450K,  pyrosequencing |
| TP3_1 | Male | 9 | Han | autism | ASD-discordant MZ twins | Illumina 450K,  pyrosequencing |
| TP4_1 | Male | 11 | Han | autism | ASD-discordant MZ twins | RRBS,  pyrosequencing |
| TP5_1 | Male | 7 | Han | autism | ASD-discordant MZ twins | RRBS,  pyrosequencing |
| TP6_1 | male | 2 | Han | autism | ASD-concordant MZ twins | pyrosequencing |
| TP7_1 | male | 3 | Han | autism | ASD-concordant MZ twins | pyrosequencing |
| TP8_1 | male | 4 | Han | autism | ASD-concordant MZ twins | pyrosequencing |
| TP9_1 | female | 4 | Han | autism | ASD-concordant MZ twins | pyrosequencing |
| TP6_2 | male | 2 | Han | autism | ASD-concordant MZ twins | pyrosequencing |
| TP7_2 | male | 3 | Han | autism | ASD-concordant MZ twins | pyrosequencing |
| TP8_2 | male | 4 | Han | autism | ASD-concordant MZ twins | pyrosequencing |
| TP9_2 | female | 4 | Han | autism | ASD-concordant MZ twins | pyrosequencing |
| 792 | male | 3 | Han | autism | sporadic | pyrosequencing |
| 794 | male | 3 | Han | autism | sporadic | pyrosequencing |
| 795 | male | 5 | Han | autism | sporadic | pyrosequencing |
| 796 | male | 4 | Han | autism | sporadic | pyrosequencing |
| 814 | male | 4 | Han | autism | sporadic | pyrosequencing |
| 819 | male | 4 | Han | autism | sporadic | pyrosequencing |
| 821 | male | 4 | Han | autism | sporadic | pyrosequencing |
| 822 | male | 4 | Han | autism | sporadic | pyrosequencing |
| 824 | male | 4 | Han | autism | sporadic | pyrosequencing |
| 825 | male | 5 | Han | autism | sporadic | pyrosequencing |
| 826 | male | 4 | Han | autism | sporadic | pyrosequencing |
| 828 | male | 4 | Han | autism | sporadic | pyrosequencing |
| 831 | male | 6 | Han | autism | sporadic | pyrosequencing |
| 833 | male | 6 | Han | autism | sporadic | pyrosequencing |
| 837 | male | 4 | Han | autism | sporadic | pyrosequencing |
| 839 | male | 4 | Han | autism | sporadic | pyrosequencing |
| 866 | male | 4 | Han | autism | sporadic | pyrosequencing |
| 871 | male | 4 | Han | autism | sporadic | pyrosequencing |
| 873 | male | 4 | Han | autism | sporadic | pyrosequencing |
| 875 | female | 4 | Han | autism | sporadic | pyrosequencing |
| CL04 | male | 5 | Han | autism | sporadic | pyrosequencing |
| CL18 | male | 5 | Han | autism | sporadic | pyrosequencing |
| CL24 | male | 5 | Han | autism | sporadic | pyrosequencing |
| CL48 | male | 5 | Han | autism | sporadic | pyrosequencing |
| CL50 | male | 6 | Han | autism | sporadic | pyrosequencing |
| CL51 | male | 4 | Han | autism | sporadic | pyrosequencing |
| CL52 | female | 6 | Han | autism | sporadic | pyrosequencing |
| CL54 | female | 4 | Han | autism | sporadic | pyrosequencing |
| CL55 | male | 4 | Han | autism | sporadic | pyrosequencing |
| CL56 | female | 6 | Han | autism | sporadic | pyrosequencing |
| **Control** |  |  |  |  |  |  |
| TP1_2 | Female | 12 | Han | control | ASD-discordant MZ twins | Illumina 450K,  pyrosequencing |
| TP2_2 | Female | 2 | Han | control | ASD-discordant MZ twins | Illumina 450K,  pyrosequencing |
| TP3_2 | Male | 9 | Han | control | ASD-discordant MZ twins | Illumina 450K,  pyrosequencing |
| TP4_2 | Male | 11 | Han | control | ASD-discordant MZ twins | RRBS,  pyrosequencing |
| TP5_2 | Male | 7 | Han | control | ASD-discordant MZ twins | RRBS,  pyrosequencing |
| DJ009 | male | 4 | Han | control | sporadic | pyrosequencing |
| DJ005 | male | 4 | Han | control | sporadic | pyrosequencing |
| DJ038 | male | 5 | Han | control | sporadic | pyrosequencing |
| DJ021 | male | 4 | Han | control | sporadic | pyrosequencing |
| XF009 | male | 4 | Han | control | sporadic | pyrosequencing |
| XF018 | male | 3 | Han | control | sporadic | pyrosequencing |
| DJ019 | male | 4 | Han | control | sporadic | pyrosequencing |
| DJ010 | male | 4 | Han | control | sporadic | pyrosequencing |
| XF014 | male | 4 | Han | control | sporadic | pyrosequencing |
| XF007 | male | 5 | Han | control | sporadic | pyrosequencing |
| DJ016 | male | 4 | Han | control | sporadic | pyrosequencing |
| DJ013 | male | 4 | Han | control | sporadic | pyrosequencing |
| DJ040 | male | 6 | Han | control | sporadic | pyrosequencing |
| DJ030 | male | 5 | Han | control | sporadic | pyrosequencing |
| DJ020 | male | 4 | Han | control | sporadic | pyrosequencing |
| DJ007 | male | 4 | Han | control | sporadic | pyrosequencing |
| FL002 | male | 4 | Han | control | sporadic | pyrosequencing |
| FL008 | male | 4 | Han | control | sporadic | pyrosequencing |
| XF023 | male | 4 | Han | control | sporadic | pyrosequencing |
| FL007 | female | 4 | Han | control | sporadic | pyrosequencing |
| DJ027 | male | 5 | Han | control | sporadic | pyrosequencing |
| DJ028 | male | 5 | Han | control | sporadic | pyrosequencing |
| DJ029 | male | 5 | Han | control | sporadic | pyrosequencing |
| XF008 | male | 5 | Han | control | sporadic | pyrosequencing |
| DJ033 | male | 6 | Han | control | sporadic | pyrosequencing |
| DJ023 | male | 4 | Han | control | sporadic | pyrosequencing |
| DJ034 | female | 6 | Han | control | sporadic | pyrosequencing |
| DJ018 | female | 4 | Han | control | sporadic | pyrosequencing |
| DJ037 | male | 4 | Han | control | sporadic | pyrosequencing |
| XF011 | female | 5 | Han | control | sporadic | pyrosequencing |

**Supplementary Table 2 2397 differential methylated intersected genes identified between the two datasets of five ASD-discordant MZ twins**

| **No.** | **Gene symbol** | **No.** | **Gene symbol** | **No.** | **Gene symbol** |
| --- | --- | --- | --- | --- | --- |
| 1 | H2A1H_HUMAN | 801 | ATS19_HUMAN | 1601 | KRA49_HUMAN |
| 2 | H2A2B_HUMAN | 802 | ASB16_HUMAN | 1602 | KRBA1_HUMAN |
| 3 | PPR1B_HUMAN | 803 | CST11_HUMAN | 1603 | LAMC1_HUMAN |
| 4 | COX7S_HUMAN | 804 | CRYL1_HUMAN | 1604 | CNDG2_HUMAN |
| 5 | HXC9_HUMAN | 805 | CRYM_HUMAN | 1605 | CEP76_HUMAN |
| 6 | HXK1_HUMAN | 806 | CT459_HUMAN | 1606 | LDHB_HUMAN |
| 7 | TMM69_HUMAN | 807 | CT47B_HUMAN | 1607 | CYLC1_HUMAN |
| 8 | TMM78_HUMAN | 808 | VEGFC_HUMAN | 1608 | CYTD_HUMAN |
| 9 | TMTC4_HUMAN | 809 | CXCL7_HUMAN | 1609 | LMIP_HUMAN |
| 10 | TBL2_HUMAN | 810 | CXL11_HUMAN | 1610 | LENG1_HUMAN |
| 11 | THNS1_HUMAN | 811 | CORO6_HUMAN | 1611 | LFG2_HUMAN |
| 12 | THOC5_HUMAN | 812 | COX19_HUMAN | 1612 | LGAT1_HUMAN |
| 13 | TIGD3_HUMAN | 813 | CRGN_HUMAN | 1613 | LCE4A_HUMAN |
| 14 | TM158_HUMAN | 814 | CRLF1_HUMAN | 1614 | LCN8_HUMAN |
| 15 | TM252_HUMAN | 815 | CRSPL_HUMAN | 1615 | ZN726_HUMAN |
| 16 | TMM31_HUMAN | 816 | CS043_HUMAN | 1616 | LMAN1_HUMAN |
| 17 | TFDP1_HUMAN | 817 | CQ112_HUMAN | 1617 | KRT38_HUMAN |
| 18 | TRYB1_HUMAN | 818 | CR021_HUMAN | 1618 | IMP1L_HUMAN |
| 19 | TSR1_HUMAN | 819 | CSF1_HUMAN | 1619 | IMPA1_HUMAN |
| 20 | TM121_HUMAN | 820 | CTU1_HUMAN | 1620 | LALBA_HUMAN |
| 21 | TM145_HUMAN | 821 | CRF_HUMAN | 1621 | LAS2_HUMAN |
| 22 | TM163_HUMAN | 822 | CRUM3_HUMAN | 1622 | LCE1E_HUMAN |
| 23 | TXLNG_HUMAN | 823 | CX024_HUMAN | 1623 | LCN1_HUMAN |
| 24 | TM1L2_HUMAN | 824 | CXE1_HUMAN | 1624 | LETM2_HUMAN |
| 25 | TX264_HUMAN | 825 | CY24B_HUMAN | 1625 | LAMA3_HUMAN |
| 26 | TMM61_HUMAN | 826 | CPLX3_HUMAN | 1626 | LRIQ3_HUMAN |
| 27 | TMPSC_HUMAN | 827 | TMM52_HUMAN | 1627 | LRIT1_HUMAN |
| 28 | RAD21_HUMAN | 828 | CP27A_HUMAN | 1628 | M10L1_HUMAN |
| 29 | TMUB1_HUMAN | 829 | CSK21_HUMAN | 1629 | M3K5_HUMAN |
| 30 | TMX3_HUMAN | 830 | CTRL_HUMAN | 1630 | LEG9C_HUMAN |
| 31 | PTN_HUMAN | 831 | CX023_HUMAN | 1631 | LIAT1_HUMAN |
| 32 | TOPZ1_HUMAN | 832 | CXB5_HUMAN | 1632 | LIMD2_HUMAN |
| 33 | TEX48_HUMAN | 833 | CUX1_HUMAN | 1633 | LIX1L_HUMAN |
| 34 | TM101_HUMAN | 834 | AP5Z1_HUMAN | 1634 | LKAM1_HUMAN |
| 35 | KKCC2_HUMAN | 835 | TRAIP_HUMAN | 1635 | LTBP2_HUMAN |
| 36 | TO6BL_HUMAN | 836 | DAAF4_HUMAN | 1636 | LV312_HUMAN |
| 37 | TPRA1_HUMAN | 837 | DCTP1_HUMAN | 1637 | LRFN4_HUMAN |
| 38 | TKN1_HUMAN | 838 | DEF6_HUMAN | 1638 | LRR70_HUMAN |
| 39 | TM38A_HUMAN | 839 | MNS1_HUMAN | 1639 | LRRN1_HUMAN |
| 40 | TMCO1_HUMAN | 840 | CXXC5_HUMAN | 1640 | LSP1_HUMAN |
| 41 | IZUM3_HUMAN | 841 | D106A_HUMAN | 1641 | LZIC_HUMAN |
| 42 | TM201_HUMAN | 842 | DCMC_HUMAN | 1642 | LZTL1_HUMAN |
| 43 | TRAD1_HUMAN | 843 | CUL9_HUMAN | 1643 | LRC23_HUMAN |
| 44 | NEUL3_HUMAN | 844 | DCA15_HUMAN | 1644 | LRP3_HUMAN |
| 45 | NEUM_HUMAN | 845 | DCAF6_HUMAN | 1645 | LRR3C_HUMAN |
| 46 | NEUR1_HUMAN | 846 | DCDC5_HUMAN | 1646 | MCSP_HUMAN |
| 47 | TOM7_HUMAN | 847 | RBTN1_HUMAN | 1647 | LOXL3_HUMAN |
| 48 | TP8L2_HUMAN | 848 | PRA14_HUMAN | 1648 | LYST_HUMAN |
| 49 | TPM3_HUMAN | 849 | RCC1_HUMAN | 1649 | M3K13_HUMAN |
| 50 | TREA_HUMAN | 850 | REEP5_HUMAN | 1650 | M3K15_HUMAN |
| 51 | TREF1_HUMAN | 851 | RELL1_HUMAN | 1651 | LS14B_HUMAN |
| 52 | TRI25_HUMAN | 852 | REV1_HUMAN | 1652 | M4A12_HUMAN |
| 53 | F19A1_HUMAN | 853 | DC1I2_HUMAN | 1653 | MB211_HUMAN |
| 54 | TMPS7_HUMAN | 854 | DCP1A_HUMAN | 1654 | MB3L2_HUMAN |
| 55 | TPIS_HUMAN | 855 | CP2D7_HUMAN | 1655 | LPXN_HUMAN |
| 56 | TOR1A_HUMAN | 856 | CQ10A_HUMAN | 1656 | CST9_HUMAN |
| 57 | TP8L3_HUMAN | 857 | EZH1_HUMAN | 1657 | M4A6A_HUMAN |
| 58 | TPP2_HUMAN | 858 | CRCM_HUMAN | 1658 | M4K2_HUMAN |
| 59 | SP4_HUMAN | 859 | CT144_HUMAN | 1659 | MA1B1_HUMAN |
| 60 | ZC21C_HUMAN | 860 | CT47A_HUMAN | 1660 | MA2B2_HUMAN |
| 61 | RAB7L_HUMAN | 861 | DAPK3_HUMAN | 1661 | LYPD5_HUMAN |
| 62 | TRM44_HUMAN | 862 | D131B_HUMAN | 1662 | LY66C_HUMAN |
| 63 | TRM7_HUMAN | 863 | DBNL_HUMAN | 1663 | LY75_HUMAN |
| 64 | TRPM6_HUMAN | 864 | DCD2C_HUMAN | 1664 | MAGA5_HUMAN |
| 65 | TRY3_HUMAN | 865 | CYYR1_HUMAN | 1665 | MADL2_HUMAN |
| 66 | TM207_HUMAN | 866 | DB113_HUMAN | 1666 | MAGAC_HUMAN |
| 67 | TM230_HUMAN | 867 | DCNL1_HUMAN | 1667 | MAJIN_HUMAN |
| 68 | TMIG2_HUMAN | 868 | DCK_HUMAN | 1668 | LCP2_HUMAN |
| 69 | TP4A2_HUMAN | 869 | SMTN_HUMAN | 1669 | LIPM_HUMAN |
| 70 | TRML2_HUMAN | 870 | DANCR_HUMAN | 1670 | LIPN_HUMAN |
| 71 | TIE1_HUMAN | 871 | DAZP2_HUMAN | 1671 | MDGA1_HUMAN |
| 72 | TLX1_HUMAN | 872 | CU037_HUMAN | 1672 | MAP6_HUMAN |
| 73 | TRY2_HUMAN | 873 | CU093_HUMAN | 1673 | PO3F1_HUMAN |
| 74 | TRDMT_HUMAN | 874 | CV039_HUMAN | 1674 | LTOR2_HUMAN |
| 75 | TSN18_HUMAN | 875 | CYB5B_HUMAN | 1675 | LT4R1_HUMAN |
| 76 | TPC6A_HUMAN | 876 | DG2L7_HUMAN | 1676 | LTMD1_HUMAN |
| 77 | TPTE_HUMAN | 877 | DGKD_HUMAN | 1677 | LYRM2_HUMAN |
| 78 | TR43B_HUMAN | 878 | DICER_HUMAN | 1678 | M4A8_HUMAN |
| 79 | TMOD3_HUMAN | 879 | DJC14_HUMAN | 1679 | LRIF1_HUMAN |
| 80 | TMTC3_HUMAN | 880 | DKC1_HUMAN | 1680 | LRN4L_HUMAN |
| 81 | TRIML_HUMAN | 881 | DKK3_HUMAN | 1681 | M2OM_HUMAN |
| 82 | TR10D_HUMAN | 882 | DMWD_HUMAN | 1682 | MA2A2_HUMAN |
| 83 | TRAF2_HUMAN | 883 | DP13B_HUMAN | 1683 | MAAI_HUMAN |
| 84 | TRAM1_HUMAN | 884 | DSN1_HUMAN | 1684 | MAF_HUMAN |
| 85 | TSYL2_HUMAN | 885 | DHE4_HUMAN | 1685 | LUZP1_HUMAN |
| 86 | TT30A_HUMAN | 886 | DMB_HUMAN | 1686 | LYRIC_HUMAN |
| 87 | TYOBP_HUMAN | 887 | DMRTB_HUMAN | 1687 | MAGI3_HUMAN |
| 88 | TYW2_HUMAN | 888 | DNLI1_HUMAN | 1688 | MAP12_HUMAN |
| 89 | UB2R2_HUMAN | 889 | CTIF_HUMAN | 1689 | MARH1_HUMAN |
| 90 | UBE2H_HUMAN | 890 | DDX41_HUMAN | 1690 | MARH6_HUMAN |
| 91 | TSAS2_HUMAN | 891 | SPI2B_HUMAN | 1691 | MARK2_HUMAN |
| 92 | TSPOB_HUMAN | 892 | SPIT3_HUMAN | 1692 | MC5R_HUMAN |
| 93 | TSPY8_HUMAN | 893 | VPP3_HUMAN | 1693 | LTOR3_HUMAN |
| 94 | TRPV2_HUMAN | 894 | DQB1_HUMAN | 1694 | LYAM1_HUMAN |
| 95 | TRPV6_HUMAN | 895 | DCR1B_HUMAN | 1695 | MAEL_HUMAN |
| 96 | LV316_HUMAN | 896 | DEDD2_HUMAN | 1696 | MAFF_HUMAN |
| 97 | MARH2_HUMAN | 897 | DG2L6_HUMAN | 1697 | MARK3_HUMAN |
| 98 | LV322_HUMAN | 898 | DKKL1_HUMAN | 1698 | LRC34_HUMAN |
| 99 | LV746_HUMAN | 899 | DNM1L_HUMAN | 1699 | MDM2_HUMAN |
| 100 | LYPA1_HUMAN | 900 | DPF3_HUMAN | 1700 | LR37B_HUMAN |
| 101 | MA2C1_HUMAN | 901 | DLG4_HUMAN | 1701 | MCFD2_HUMAN |
| 102 | MAGI2_HUMAN | 902 | DNJA4_HUMAN | 1702 | HMCS1_HUMAN |
| 103 | MAG_HUMAN | 903 | EST4A_HUMAN | 1703 | MCMD2_HUMAN |
| 104 | MANF_HUMAN | 904 | ESTD_HUMAN | 1704 | MEG10_HUMAN |
| 105 | LSME1_HUMAN | 905 | METK2_HUMAN | 1705 | MENT_HUMAN |
| 106 | U633B_HUMAN | 906 | M3K21_HUMAN | 1706 | MEOX2_HUMAN |
| 107 | UCP3_HUMAN | 907 | DB118_HUMAN | 1707 | MFGM_HUMAN |
| 108 | TTL13_HUMAN | 908 | DSG2_HUMAN | 1708 | MIC25_HUMAN |
| 109 | U17L8_HUMAN | 909 | DTX3_HUMAN | 1709 | MED18_HUMAN |
| 110 | U2AF2_HUMAN | 910 | DUS7_HUMAN | 1710 | MED24_HUMAN |
| 111 | TPC1_HUMAN | 911 | EDC3_HUMAN | 1711 | MCF2L_HUMAN |
| 112 | TRML4_HUMAN | 912 | EF1B_HUMAN | 1712 | MET2A_HUMAN |
| 113 | TR10A_HUMAN | 913 | EFGM_HUMAN | 1713 | MCHR1_HUMAN |
| 114 | TWF2_HUMAN | 914 | EID2_HUMAN | 1714 | MDFI_HUMAN |
| 115 | TTC4_HUMAN | 915 | EIF3E_HUMAN | 1715 | MED22_HUMAN |
| 116 | TTI2_HUMAN | 916 | DESI1_HUMAN | 1716 | MEG11_HUMAN |
| 117 | TTC23_HUMAN | 917 | DEUP1_HUMAN | 1717 | MEGF6_HUMAN |
| 118 | TPGS2_HUMAN | 918 | DHRS9_HUMAN | 1718 | MELPH_HUMAN |
| 119 | TRI48_HUMAN | 919 | DHCR7_HUMAN | 1719 | MEP1B_HUMAN |
| 120 | UTP11_HUMAN | 920 | DHE3_HUMAN | 1720 | MESP2_HUMAN |
| 121 | UTY_HUMAN | 921 | DIEXF_HUMAN | 1721 | MEX3A_HUMAN |
| 122 | VBPC1_HUMAN | 922 | DLX3_HUMAN | 1722 | MEX3C_HUMAN |
| 123 | UBP32_HUMAN | 923 | DMP46_HUMAN | 1723 | MF14A_HUMAN |
| 124 | VCX2_HUMAN | 924 | DEOC_HUMAN | 1724 | MDM4_HUMAN |
| 125 | UBXN7_HUMAN | 925 | B3A2_HUMAN | 1725 | MCM6_HUMAN |
| 126 | VAS1_HUMAN | 926 | B3A3_HUMAN | 1726 | MEA1_HUMAN |
| 127 | GRIK2_HUMAN | 927 | BAF_HUMAN | 1727 | MAGC1_HUMAN |
| 128 | TZAP_HUMAN | 928 | DNLI4_HUMAN | 1728 | MAPK5_HUMAN |
| 129 | TM253_HUMAN | 929 | EI2BB_HUMAN | 1729 | HV349_HUMAN |
| 130 | U730_HUMAN | 930 | DNJ5B_HUMAN | 1730 | MAT2B_HUMAN |
| 131 | UBAC2_HUMAN | 931 | DNJC5_HUMAN | 1731 | MBOA7_HUMAN |
| 132 | UVRAG_HUMAN | 932 | DOPP1_HUMAN | 1732 | MCRS1_HUMAN |
| 133 | UB2Q1_HUMAN | 933 | CSKP_HUMAN | 1733 | MCTP1_HUMAN |
| 134 | UBFD1_HUMAN | 934 | DRB3_HUMAN | 1734 | SMS1_HUMAN |
| 135 | UCHL1_HUMAN | 935 | ELB3B_HUMAN | 1735 | MUSK_HUMAN |
| 136 | USP9X_HUMAN | 936 | EIF1B_HUMAN | 1736 | MYOTI_HUMAN |
| 137 | TX101_HUMAN | 937 | DUPD1_HUMAN | 1737 | MITF_HUMAN |
| 138 | TXNL1_HUMAN | 938 | EMX1_HUMAN | 1738 | MK09_HUMAN |
| 139 | SNUFL_HUMAN | 939 | ELK4_HUMAN | 1739 | MLX_HUMAN |
| 140 | SPR2A_HUMAN | 940 | DRD1_HUMAN | 1740 | MMP17_HUMAN |
| 141 | SESQ2_HUMAN | 941 | DSG1_HUMAN | 1741 | M4A14_HUMAN |
| 142 | SPAC7_HUMAN | 942 | EFNB1_HUMAN | 1742 | MATN1_HUMAN |
| 143 | UBP31_HUMAN | 943 | EFNB3_HUMAN | 1743 | NAA20_HUMAN |
| 144 | UBP3_HUMAN | 944 | DONS_HUMAN | 1744 | MRM2_HUMAN |
| 145 | UBP2_HUMAN | 945 | DPA1_HUMAN | 1745 | MSD3_HUMAN |
| 146 | UCN3_HUMAN | 946 | DPOLM_HUMAN | 1746 | MSI2H_HUMAN |
| 147 | UN45B_HUMAN | 947 | DYH14_HUMAN | 1747 | MSPD2_HUMAN |
| 148 | TVA1_HUMAN | 948 | DP13A_HUMAN | 1748 | MY18B_HUMAN |
| 149 | UCP4_HUMAN | 949 | DPH6_HUMAN | 1749 | MRGX3_HUMAN |
| 150 | UEVLD_HUMAN | 950 | DPOD1_HUMAN | 1750 | MRP2_HUMAN |
| 151 | UBN1_HUMAN | 951 | LRC39_HUMAN | 1751 | MS4A5_HUMAN |
| 152 | UBP12_HUMAN | 952 | DUS1L_HUMAN | 1752 | MTMRD_HUMAN |
| 153 | UIF_HUMAN | 953 | DYL2_HUMAN | 1753 | MTURN_HUMAN |
| 154 | UN13A_HUMAN | 954 | EBP2_HUMAN | 1754 | FBX28_HUMAN |
| 155 | UBA1L_HUMAN | 955 | ECM2_HUMAN | 1755 | FBX34_HUMAN |
| 156 | UBE2A_HUMAN | 956 | ELB3D_HUMAN | 1756 | FCG2B_HUMAN |
| 157 | UBE2S_HUMAN | 957 | EDNRB_HUMAN | 1757 | FDFT_HUMAN |
| 158 | UNC5C_HUMAN | 958 | DUS16_HUMAN | 1758 | FEV_HUMAN |
| 159 | UNKL_HUMAN | 959 | DTD2_HUMAN | 1759 | MT1A_HUMAN |
| 160 | UNC5D_HUMAN | 960 | EGFLA_HUMAN | 1760 | AP2A2_HUMAN |
| 161 | VAV_HUMAN | 961 | ENOL_HUMAN | 1761 | MTUS1_HUMAN |
| 162 | UPK2_HUMAN | 962 | EPC2_HUMAN | 1762 | MT3_HUMAN |
| 163 | UPKL2_HUMAN | 963 | EFCB8_HUMAN | 1763 | MTF2_HUMAN |
| 164 | UR2R_HUMAN | 964 | EFNA4_HUMAN | 1764 | MVP_HUMAN |
| 165 | URM1_HUMAN | 965 | EGR1_HUMAN | 1765 | MSMP_HUMAN |
| 166 | VGLL3_HUMAN | 966 | EI2BA_HUMAN | 1766 | MYT1L_HUMAN |
| 167 | VN1R3_HUMAN | 967 | EME1_HUMAN | 1767 | MUC5A_HUMAN |
| 168 | JOS1_HUMAN | 968 | DYH1_HUMAN | 1768 | MT21E_HUMAN |
| 169 | UDB28_HUMAN | 969 | NPS3A_HUMAN | 1769 | MYBA_HUMAN |
| 170 | UROK_HUMAN | 970 | EOMES_HUMAN | 1770 | MYH11_HUMAN |
| 171 | VAMP8_HUMAN | 971 | ERFE_HUMAN | 1771 | MYH8_HUMAN |
| 172 | TYRP1_HUMAN | 972 | ERG24_HUMAN | 1772 | MYO16_HUMAN |
| 173 | TYY1_HUMAN | 973 | EPCAM_HUMAN | 1773 | NBR1_HUMAN |
| 174 | U17L5_HUMAN | 974 | ERF1_HUMAN | 1774 | MUM1_HUMAN |
| 175 | TYW1_HUMAN | 975 | ESX1_HUMAN | 1775 | MYCP1_HUMAN |
| 176 | VHL_HUMAN | 976 | EXOC1_HUMAN | 1776 | NDUB8_HUMAN |
| 177 | VMA5A_HUMAN | 977 | EXOC2_HUMAN | 1777 | NECT1_HUMAN |
| 178 | V2R_HUMAN | 978 | EXOSX_HUMAN | 1778 | NDK8_HUMAN |
| 179 | VPREB_HUMAN | 979 | DSPP_HUMAN | 1779 | NOM1_HUMAN |
| 180 | VPS11_HUMAN | 980 | ELOV1_HUMAN | 1780 | NDF6_HUMAN |
| 181 | VPS54_HUMAN | 981 | ELP5_HUMAN | 1781 | NDUA4_HUMAN |
| 182 | VS10L_HUMAN | 982 | EMD_HUMAN | 1782 | HMR1_HUMAN |
| 183 | VSIG1_HUMAN | 983 | ERR3_HUMAN | 1783 | NET4_HUMAN |
| 184 | WASH2_HUMAN | 984 | ESRP1_HUMAN | 1784 | NKX25_HUMAN |
| 185 | WDFY3_HUMAN | 985 | ENY2_HUMAN | 1785 | NDUV2_HUMAN |
| 186 | VCX3_HUMAN | 986 | EP400_HUMAN | 1786 | NT5M_HUMAN |
| 187 | VPK18_HUMAN | 987 | EPIPL_HUMAN | 1787 | NTM2D_HUMAN |
| 188 | WIZ_HUMAN | 988 | EPS8_HUMAN | 1788 | NRX1A_HUMAN |
| 189 | WRB_HUMAN | 989 | ERC6L_HUMAN | 1789 | NRX2A_HUMAN |
| 190 | XRCC2_HUMAN | 990 | ERG28_HUMAN | 1790 | NEUA_HUMAN |
| 191 | VPS18_HUMAN | 991 | CFA53_HUMAN | 1791 | NDUA9_HUMAN |
| 192 | UBXN6_HUMAN | 992 | EVA1C_HUMAN | 1792 | NGN1_HUMAN |
| 193 | WNT7B_HUMAN | 993 | EVPL_HUMAN | 1793 | NR2CA_HUMAN |
| 194 | XIRP2_HUMAN | 994 | EBLN1_HUMAN | 1794 | NELFA_HUMAN |
| 195 | VPRE3_HUMAN | 995 | ECT2L_HUMAN | 1795 | NOP2_HUMAN |
| 196 | VGLL1_HUMAN | 996 | ELOC_HUMAN | 1796 | NR6A1_HUMAN |
| 197 | WDR5B_HUMAN | 997 | EMAL5_HUMAN | 1797 | NSN5B_HUMAN |
| 198 | VGLU1_HUMAN | 998 | EMC10_HUMAN | 1798 | NCDN_HUMAN |
| 199 | VNN2_HUMAN | 999 | ENH1_HUMAN | 1799 | NCF4_HUMAN |
| 200 | WNT11_HUMAN | 1000 | ENK19_HUMAN | 1800 | NCKPL_HUMAN |
| 201 | WISP2_HUMAN | 1001 | ENKD1_HUMAN | 1801 | NCK1_HUMAN |
| 202 | WDR75_HUMAN | 1002 | EFCB2_HUMAN | 1802 | NCOR2_HUMAN |
| 203 | K1C25_HUMAN | 1003 | EFCB5_HUMAN | 1803 | NLGN2_HUMAN |
| 204 | CAPON_HUMAN | 1004 | EHBP1_HUMAN | 1804 | NMS_HUMAN |
| 205 | ACHB2_HUMAN | 1005 | EI2BE_HUMAN | 1805 | NKTR_HUMAN |
| 206 | NPHP1_HUMAN | 1006 | ERI6B_HUMAN | 1806 | MYCN_HUMAN |
| 207 | ACO11_HUMAN | 1007 | ERO1B_HUMAN | 1807 | NPM3_HUMAN |
| 208 | ACSM4_HUMAN | 1008 | F231B_HUMAN | 1808 | NKX22_HUMAN |
| 209 | AB17B_HUMAN | 1009 | ERD23_HUMAN | 1809 | NKX28_HUMAN |
| 210 | RL38_HUMAN | 1010 | EREG_HUMAN | 1810 | NKX32_HUMAN |
| 211 | A1CF_HUMAN | 1011 | ERPG3_HUMAN | 1811 | NHLC2_HUMAN |
| 212 | ORML2_HUMAN | 1012 | F210A_HUMAN | 1812 | PHLB1_HUMAN |
| 213 | AATF_HUMAN | 1013 | F213A_HUMAN | 1813 | PHLB2_HUMAN |
| 214 | CU062_HUMAN | 1014 | FA53C_HUMAN | 1814 | NLRC4_HUMAN |
| 215 | RL19_HUMAN | 1015 | FA7_HUMAN | 1815 | NNRE_HUMAN |
| 216 | ADAM5_HUMAN | 1016 | FA89A_HUMAN | 1816 | NOC3L_HUMAN |
| 217 | AFG1L_HUMAN | 1017 | FA9_HUMAN | 1817 | NMD3A_HUMAN |
| 218 | APEL_HUMAN | 1018 | FAF1_HUMAN | 1818 | NOXO1_HUMAN |
| 219 | UST_HUMAN | 1019 | FAS_HUMAN | 1819 | PIDD1_HUMAN |
| 220 | AHI1_HUMAN | 1020 | FBH1_HUMAN | 1820 | PP4R2_HUMAN |
| 221 | AK1A1_HUMAN | 1021 | FBX25_HUMAN | 1821 | NPIB5_HUMAN |
| 222 | ARMX1_HUMAN | 1022 | FBX44_HUMAN | 1822 | NDUBA_HUMAN |
| 223 | FOXE1_HUMAN | 1023 | ETAA1_HUMAN | 1823 | NPY_HUMAN |
| 224 | HMOX2_HUMAN | 1024 | FA98C_HUMAN | 1824 | FAM3D_HUMAN |
| 225 | AP3D1_HUMAN | 1025 | FABP7_HUMAN | 1825 | NQO2_HUMAN |
| 226 | DHX37_HUMAN | 1026 | FA43B_HUMAN | 1826 | NFM_HUMAN |
| 227 | RIPK2_HUMAN | 1027 | FA57A_HUMAN | 1827 | NP1L1_HUMAN |
| 228 | YE016_HUMAN | 1028 | ETBR2_HUMAN | 1828 | NPAL2_HUMAN |
| 229 | HINT3_HUMAN | 1029 | EVI5_HUMAN | 1829 | NR2F6_HUMAN |
| 230 | XRCC3_HUMAN | 1030 | F13B_HUMAN | 1830 | NELFB_HUMAN |
| 231 | XPP3_HUMAN | 1031 | F193B_HUMAN | 1831 | NEMF_HUMAN |
| 232 | PI3R4_HUMAN | 1032 | F221B_HUMAN | 1832 | NEP_HUMAN |
| 233 | ANR53_HUMAN | 1033 | F90AR_HUMAN | 1833 | NFAM1_HUMAN |
| 234 | AP2A1_HUMAN | 1034 | FA10_HUMAN | 1834 | NFE4_HUMAN |
| 235 | VSXL2_HUMAN | 1035 | FA156_HUMAN | 1835 | NUPR2_HUMAN |
| 236 | ATPB_HUMAN | 1036 | FA24A_HUMAN | 1836 | TKN4_HUMAN |
| 237 | VWA1_HUMAN | 1037 | FA53A_HUMAN | 1837 | O52R1_HUMAN |
| 238 | WDFY1_HUMAN | 1038 | FA83D_HUMAN | 1838 | NKG2A_HUMAN |
| 239 | ZNT7_HUMAN | 1039 | F131C_HUMAN | 1839 | NOTC1_HUMAN |
| 240 | NOV_HUMAN | 1040 | F240B_HUMAN | 1840 | NPIB3_HUMAN |
| 241 | ZN701_HUMAN | 1041 | ETV6_HUMAN | 1841 | O10H4_HUMAN |
| 242 | CLD34_HUMAN | 1042 | EXOC3_HUMAN | 1842 | O11H1_HUMAN |
| 243 | ZNT1_HUMAN | 1043 | EXOS8_HUMAN | 1843 | NPT2A_HUMAN |
| 244 | ZO2_HUMAN | 1044 | F153A_HUMAN | 1844 | CXCL5_HUMAN |
| 245 | ZSA5A_HUMAN | 1045 | PTTG1_HUMAN | 1845 | NPY5R_HUMAN |
| 246 | F177B_HUMAN | 1046 | F157B_HUMAN | 1846 | NRX3A_HUMAN |
| 247 | ZN596_HUMAN | 1047 | EFCC1_HUMAN | 1847 | O51F2_HUMAN |
| 248 | ZCPW2_HUMAN | 1048 | ZN770_HUMAN | 1848 | O6C76_HUMAN |
| 249 | ZSC23_HUMAN | 1049 | F228A_HUMAN | 1849 | NSGX_HUMAN |
| 250 | ZSC10_HUMAN | 1050 | VIME_HUMAN | 1850 | NUP54_HUMAN |
| 251 | WWP1_HUMAN | 1051 | ELNE_HUMAN | 1851 | O10T2_HUMAN |
| 252 | RL32_HUMAN | 1052 | CFA58_HUMAN | 1852 | O10W1_HUMAN |
| 253 | YIF1B_HUMAN | 1053 | FA32A_HUMAN | 1853 | O51A4_HUMAN |
| 254 | YH009_HUMAN | 1054 | FA46D_HUMAN | 1854 | O11L1_HUMAN |
| 255 | YIPF2_HUMAN | 1055 | ZN799_HUMAN | 1855 | O52N5_HUMAN |
| 256 | YK026_HUMAN | 1056 | FA83H_HUMAN | 1856 | ODBB_HUMAN |
| 257 | YJ004_HUMAN | 1057 | FLVC2_HUMAN | 1857 | NDK5_HUMAN |
| 258 | YQ048_HUMAN | 1058 | F262_HUMAN | 1858 | NDUB1_HUMAN |
| 259 | YPEL5_HUMAN | 1059 | F263_HUMAN | 1859 | NDUF4_HUMAN |
| 260 | YQ014_HUMAN | 1060 | FAIM3_HUMAN | 1860 | NDUS5_HUMAN |
| 261 | YB035_HUMAN | 1061 | F86JP_HUMAN | 1861 | HCAR1_HUMAN |
| 262 | YIF1A_HUMAN | 1062 | FAM9B_HUMAN | 1862 | HEPN1_HUMAN |
| 263 | YD021_HUMAN | 1063 | FANCM_HUMAN | 1863 | HERC6_HUMAN |
| 264 | YS039_HUMAN | 1064 | FA30A_HUMAN | 1864 | SIA8F_HUMAN |
| 265 | ABRAL_HUMAN | 1065 | FABP5_HUMAN | 1865 | C2AS1_HUMAN |
| 266 | 2B1A_HUMAN | 1066 | FEN1_HUMAN | 1866 | NDUV1_HUMAN |
| 267 | YT001_HUMAN | 1067 | FKB14_HUMAN | 1867 | NBPF1_HUMAN |
| 268 | 3BP2_HUMAN | 1068 | FA53B_HUMAN | 1868 | NBPFF_HUMAN |
| 269 | 3HIDH_HUMAN | 1069 | F236C_HUMAN | 1869 | ABEC4_HUMAN |
| 270 | ZEP1_HUMAN | 1070 | F86B2_HUMAN | 1870 | GRIK4_HUMAN |
| 271 | YS001_HUMAN | 1071 | F91A2_HUMAN | 1871 | MYOZ2_HUMAN |
| 272 | Z658B_HUMAN | 1072 | FA24B_HUMAN | 1872 | MYP2_HUMAN |
| 273 | ZBED6_HUMAN | 1073 | FA66E_HUMAN | 1873 | MYLK4_HUMAN |
| 274 | MPRI_HUMAN | 1074 | F208B_HUMAN | 1874 | NAF1_HUMAN |
| 275 | ZN235_HUMAN | 1075 | F180A_HUMAN | 1875 | NALP5_HUMAN |
| 276 | ZN319_HUMAN | 1076 | FBLL1_HUMAN | 1876 | NCBP2_HUMAN |
| 277 | ZN467_HUMAN | 1077 | FBSL_HUMAN | 1877 | NCF1B_HUMAN |
| 278 | EF2_HUMAN | 1078 | FA84B_HUMAN | 1878 | DECR2_HUMAN |
| 279 | ZN574_HUMAN | 1079 | FAKD5_HUMAN | 1879 | TBC3G_HUMAN |
| 280 | ANXA3_HUMAN | 1080 | FAM3C_HUMAN | 1880 | SMS_HUMAN |
| 281 | DAPK1_HUMAN | 1081 | ELA_HUMAN | 1881 | TIM10_HUMAN |
| 282 | ZN527_HUMAN | 1082 | EIF3M_HUMAN | 1882 | OASL_HUMAN |
| 283 | ZN549_HUMAN | 1083 | GA113_HUMAN | 1883 | MYMX_HUMAN |
| 284 | ZN551_HUMAN | 1084 | FSCB_HUMAN | 1884 | MYMK_HUMAN |
| 285 | WDR59_HUMAN | 1085 | FXYD3_HUMAN | 1885 | MYO1G_HUMAN |
| 286 | ZN586_HUMAN | 1086 | FZD1_HUMAN | 1886 | MYOM1_HUMAN |
| 287 | NSG1_HUMAN | 1087 | FZD3_HUMAN | 1887 | MYOM2_HUMAN |
| 288 | WSB1_HUMAN | 1088 | GABPA_HUMAN | 1888 | MYPC2_HUMAN |
| 289 | GSDMB_HUMAN | 1089 | FGD5_HUMAN | 1889 | CS012_HUMAN |
| 290 | ZN816_HUMAN | 1090 | FOCAD_HUMAN | 1890 | NBPF3_HUMAN |
| 291 | Z280A_HUMAN | 1091 | FKB9L_HUMAN | 1891 | NAA40_HUMAN |
| 292 | Z286A_HUMAN | 1092 | FOXB2_HUMAN | 1892 | NACAM_HUMAN |
| 293 | ZN618_HUMAN | 1093 | FBLN7_HUMAN | 1893 | NAA16_HUMAN |
| 294 | ZEB2_HUMAN | 1094 | FBXL6_HUMAN | 1894 | AQP3_HUMAN |
| 295 | ZHX3_HUMAN | 1095 | FCER2_HUMAN | 1895 | DHX8_HUMAN |
| 296 | ZN628_HUMAN | 1096 | FL2D_HUMAN | 1896 | NAKD2_HUMAN |
| 297 | ZN75D_HUMAN | 1097 | FLT3_HUMAN | 1897 | NALP7_HUMAN |
| 298 | ZN840_HUMAN | 1098 | FX4L5_HUMAN | 1898 | NDEL1_HUMAN |
| 299 | ZN845_HUMAN | 1099 | MPP2_HUMAN | 1899 | O5AL1_HUMAN |
| 300 | BHA15_HUMAN | 1100 | GPR83_HUMAN | 1900 | PIGQ_HUMAN |
| 301 | ZNF30_HUMAN | 1101 | FTO_HUMAN | 1901 | PITX3_HUMAN |
| 302 | ZNF56_HUMAN | 1102 | FUT1_HUMAN | 1902 | PIWL4_HUMAN |
| 303 | ZN556_HUMAN | 1103 | FNDC8_HUMAN | 1903 | NKAI2_HUMAN |
| 304 | ZN565_HUMAN | 1104 | FMNL3_HUMAN | 1904 | NMNA2_HUMAN |
| 305 | YM012_HUMAN | 1105 | FXDC2_HUMAN | 1905 | KI16B_HUMAN |
| 306 | ZC4H2_HUMAN | 1106 | RN148_HUMAN | 1906 | NPIA7_HUMAN |
| 307 | ZN611_HUMAN | 1107 | GALT2_HUMAN | 1907 | NRG2_HUMAN |
| 308 | ZN625_HUMAN | 1108 | GANC_HUMAN | 1908 | NRK_HUMAN |
| 309 | ZN704_HUMAN | 1109 | CA131_HUMAN | 1909 | NS1BP_HUMAN |
| 310 | ZNF32_HUMAN | 1110 | GBG2_HUMAN | 1910 | NOSIP_HUMAN |
| 311 | ZNF35_HUMAN | 1111 | GBRR1_HUMAN | 1911 | PCD20_HUMAN |
| 312 | RAI1_HUMAN | 1112 | GBG11_HUMAN | 1912 | PCD16_HUMAN |
| 313 | RAP1B_HUMAN | 1113 | GC224_HUMAN | 1913 | PCD23_HUMAN |
| 314 | 1B46_HUMAN | 1114 | FXYD7_HUMAN | 1914 | NSDHL_HUMAN |
| 315 | ZN740_HUMAN | 1115 | GAB2_HUMAN | 1915 | NPS_HUMAN |
| 316 | YV020_HUMAN | 1116 | LUR1L_HUMAN | 1916 | NOMO1_HUMAN |
| 317 | ZBT44_HUMAN | 1117 | GALT7_HUMAN | 1917 | CAND2_HUMAN |
| 318 | KRBX4_HUMAN | 1118 | GDPD4_HUMAN | 1918 | NTM2A_HUMAN |
| 319 | HORN_HUMAN | 1119 | FOSL1_HUMAN | 1919 | O4A16_HUMAN |
| 320 | WNK1_HUMAN | 1120 | GGH_HUMAN | 1920 | PCDB9_HUMAN |
| 321 | ZBTB1_HUMAN | 1121 | GAG10_HUMAN | 1921 | TRIM5_HUMAN |
| 322 | ZACN_HUMAN | 1122 | RNH2A_HUMAN | 1922 | PHAR1_HUMAN |
| 323 | YJ001_HUMAN | 1123 | GDS1_HUMAN | 1923 | PDCD1_HUMAN |
| 324 | YK041_HUMAN | 1124 | TNIP2_HUMAN | 1924 | PDK1L_HUMAN |
| 325 | YF002_HUMAN | 1125 | TNMD_HUMAN | 1925 | PHKG2_HUMAN |
| 326 | ZN133_HUMAN | 1126 | TOB1_HUMAN | 1926 | NOSTN_HUMAN |
| 327 | ZN148_HUMAN | 1127 | GL8D2_HUMAN | 1927 | PIT1_HUMAN |
| 328 | ZN251_HUMAN | 1128 | KCNA1_HUMAN | 1928 | PKHH1_HUMAN |
| 329 | ACAD9_HUMAN | 1129 | GATB_HUMAN | 1929 | PDCD5_HUMAN |
| 330 | ZN217_HUMAN | 1130 | GBRB1_HUMAN | 1930 | PED1A_HUMAN |
| 331 | ZN254_HUMAN | 1131 | GBRB3_HUMAN | 1931 | PEN2_HUMAN |
| 332 | ZN347_HUMAN | 1132 | GLGB_HUMAN | 1932 | PGH2_HUMAN |
| 333 | ZN304_HUMAN | 1133 | GLI1_HUMAN | 1933 | PDILT_HUMAN |
| 334 | ZN428_HUMAN | 1134 | FSIP1_HUMAN | 1934 | PDLI7_HUMAN |
| 335 | ZN496_HUMAN | 1135 | FUND1_HUMAN | 1935 | PEO1_HUMAN |
| 336 | ZN517_HUMAN | 1136 | GAG2E_HUMAN | 1936 | PGBM_HUMAN |
| 337 | ZN521_HUMAN | 1137 | GPR18_HUMAN | 1937 | HXB9_HUMAN |
| 338 | ZN563_HUMAN | 1138 | GGNB1_HUMAN | 1938 | POC1B_HUMAN |
| 339 | Z385B_HUMAN | 1139 | GLIS3_HUMAN | 1939 | SND1_HUMAN |
| 340 | ZBT7B_HUMAN | 1140 | GCN1_HUMAN | 1940 | BL1S1_HUMAN |
| 341 | ZBTB2_HUMAN | 1141 | GCP4_HUMAN | 1941 | PP6R2_HUMAN |
| 342 | ZFY21_HUMAN | 1142 | GCP6_HUMAN | 1942 | PCNT_HUMAN |
| 343 | ZFYV1_HUMAN | 1143 | RP25L_HUMAN | 1943 | PDCD6_HUMAN |
| 344 | ZIC1_HUMAN | 1144 | GLDN_HUMAN | 1944 | PDE6D_HUMAN |
| 345 | ZN100_HUMAN | 1145 | FMR1N_HUMAN | 1945 | PELP1_HUMAN |
| 346 | ZN117_HUMAN | 1146 | GLP2R_HUMAN | 1946 | PEX10_HUMAN |
| 347 | ZN124_HUMAN | 1147 | GPX8_HUMAN | 1947 | PER1_HUMAN |
| 348 | ZN222_HUMAN | 1148 | GCM2_HUMAN | 1948 | PININ_HUMAN |
| 349 | ZN232_HUMAN | 1149 | GDIR1_HUMAN | 1949 | PI4KA_HUMAN |
| 350 | ZN283_HUMAN | 1150 | GHITM_HUMAN | 1950 | PI4KB_HUMAN |
| 351 | ZN320_HUMAN | 1151 | GHR_HUMAN | 1951 | PIAS1_HUMAN |
| 352 | ZN729_HUMAN | 1152 | GUC1A_HUMAN | 1952 | PLD3A_HUMAN |
| 353 | ZN772_HUMAN | 1153 | GUC2B_HUMAN | 1953 | HEN2_HUMAN |
| 354 | ADAS1_HUMAN | 1154 | GRM2A_HUMAN | 1954 | PEPA5_HUMAN |
| 355 | ZN440_HUMAN | 1155 | GRN_HUMAN | 1955 | PFKAL_HUMAN |
| 356 | ZN454_HUMAN | 1156 | GLNA_HUMAN | 1956 | PAXI1_HUMAN |
| 357 | GRM5_HUMAN | 1157 | GLRA3_HUMAN | 1957 | RHG40_HUMAN |
| 358 | ACSA_HUMAN | 1158 | GFPT2_HUMAN | 1958 | PCDGG_HUMAN |
| 359 | ACTT3_HUMAN | 1159 | GG12G_HUMAN | 1959 | PCDGH_HUMAN |
| 360 | ACV1C_HUMAN | 1160 | GPR61_HUMAN | 1960 | PCDGI_HUMAN |
| 361 | ACVR1_HUMAN | 1161 | GOG8H_HUMAN | 1961 | PCX2_HUMAN |
| 362 | 5HT2B_HUMAN | 1162 | GOGB1_HUMAN | 1962 | PKP2_HUMAN |
| 363 | 1A80_HUMAN | 1163 | GPR87_HUMAN | 1963 | PHIPL_HUMAN |
| 364 | 5NTC_HUMAN | 1164 | GPTC8_HUMAN | 1964 | PHIP_HUMAN |
| 365 | TEAD4_HUMAN | 1165 | HIG2B_HUMAN | 1965 | PILRA_HUMAN |
| 366 | ACS2A_HUMAN | 1166 | HLAE_HUMAN | 1966 | PLD1_HUMAN |
| 367 | ACL7A_HUMAN | 1167 | GHC1_HUMAN | 1967 | PLPR2_HUMAN |
| 368 | AAR2_HUMAN | 1168 | GIPC1_HUMAN | 1968 | PO2F3_HUMAN |
| 369 | ANKAR_HUMAN | 1169 | GLT13_HUMAN | 1969 | PPIL1_HUMAN |
| 370 | ACTBL_HUMAN | 1170 | GLUCM_HUMAN | 1970 | PRAM1_HUMAN |
| 371 | ACSM3_HUMAN | 1171 | GMFG_HUMAN | 1971 | PM2P5_HUMAN |
| 372 | AGAP4_HUMAN | 1172 | GMPPA_HUMAN | 1972 | PLS2_HUMAN |
| 373 | 1A02_HUMAN | 1173 | GSDMA_HUMAN | 1973 | PO2F1_HUMAN |
| 374 | SARDH_HUMAN | 1174 | HNRPU_HUMAN | 1974 | PO2F2_HUMAN |
| 375 | ACADS_HUMAN | 1175 | HIRP3_HUMAN | 1975 | POP1_HUMAN |
| 376 | F136A_HUMAN | 1176 | HLAG_HUMAN | 1976 | ARP2_HUMAN |
| 377 | ACL10_HUMAN | 1177 | GSTM2_HUMAN | 1977 | PJA2_HUMAN |
| 378 | ACHE_HUMAN | 1178 | GPR21_HUMAN | 1978 | PLST_HUMAN |
| 379 | ABCA8_HUMAN | 1179 | H10_HUMAN | 1979 | PLXB2_HUMAN |
| 380 | ABCC8_HUMAN | 1180 | PP1R8_HUMAN | 1980 | POMP_HUMAN |
| 381 | ABCF1_HUMAN | 1181 | GNAS3_HUMAN | 1981 | O14A2_HUMAN |
| 382 | ABCG5_HUMAN | 1182 | PL8L1_HUMAN | 1982 | O52B4_HUMAN |
| 383 | ABCG8_HUMAN | 1183 | PLAC9_HUMAN | 1983 | O2T12_HUMAN |
| 384 | ACES_HUMAN | 1184 | GP152_HUMAN | 1984 | O4F17_HUMAN |
| 385 | ABHD4_HUMAN | 1185 | GP157_HUMAN | 1985 | PPM1L_HUMAN |
| 386 | ABCBB_HUMAN | 1186 | CSRN2_HUMAN | 1986 | PPIE_HUMAN |
| 387 | 1A23_HUMAN | 1187 | GPN1_HUMAN | 1987 | PLIN1_HUMAN |
| 388 | 5HT3A_HUMAN | 1188 | NCS1_HUMAN | 1988 | PRA22_HUMAN |
| 389 | AN18B_HUMAN | 1189 | GPT_HUMAN | 1989 | PRD12_HUMAN |
| 390 | A4_HUMAN | 1190 | H2BFS_HUMAN | 1990 | PLIN3_HUMAN |
| 391 | ANR28_HUMAN | 1191 | GVQW1_HUMAN | 1991 | PLPP1_HUMAN |
| 392 | ABCA3_HUMAN | 1192 | H2AJ_HUMAN | 1992 | PMEL_HUMAN |
| 393 | ABCD4_HUMAN | 1193 | GSK3A_HUMAN | 1993 | POTED_HUMAN |
| 394 | 2B11_HUMAN | 1194 | H2AX_HUMAN | 1994 | PKHG6_HUMAN |
| 395 | 3BP1_HUMAN | 1195 | GRHL2_HUMAN | 1995 | PHP14_HUMAN |
| 396 | AF17_HUMAN | 1196 | CA232_HUMAN | 1996 | PIFO_HUMAN |
| 397 | ADCK2_HUMAN | 1197 | GPT2L_HUMAN | 1997 | PLDX1_HUMAN |
| 398 | AKTIP_HUMAN | 1198 | GRK6_HUMAN | 1998 | PI2R_HUMAN |
| 399 | ALG14_HUMAN | 1199 | GRP3_HUMAN | 1999 | PKN1_HUMAN |
| 400 | ALG5_HUMAN | 1200 | GS1L2_HUMAN | 2000 | PLBL1_HUMAN |
| 401 | AP1M2_HUMAN | 1201 | GRIK5_HUMAN | 2001 | PLBL2_HUMAN |
| 402 | AIFM2_HUMAN | 1202 | GTPBA_HUMAN | 2002 | POK18_HUMAN |
| 403 | ALG12_HUMAN | 1203 | HAP1_HUMAN | 2003 | PHF6_HUMAN |
| 404 | AMMR1_HUMAN | 1204 | CC187_HUMAN | 2004 | PKHF2_HUMAN |
| 405 | ALLC_HUMAN | 1205 | HDAC5_HUMAN | 2005 | PKHM2_HUMAN |
| 406 | AMPD2_HUMAN | 1206 | CDN1B_HUMAN | 2006 | PR40A_HUMAN |
| 407 | ANGT_HUMAN | 1207 | GLTL5_HUMAN | 2007 | FANCI_HUMAN |
| 408 | AK1D1_HUMAN | 1208 | GLU2B_HUMAN | 2008 | PRC1_HUMAN |
| 409 | ANDR_HUMAN | 1209 | HV601_HUMAN | 2009 | PRM2_HUMAN |
| 410 | AL4A1_HUMAN | 1210 | GP174_HUMAN | 2010 | PRORY_HUMAN |
| 411 | ANR66_HUMAN | 1211 | GSHR_HUMAN | 2011 | PLCG1_HUMAN |
| 412 | ANXA4_HUMAN | 1212 | GT2D1_HUMAN | 2012 | PINL_HUMAN |
| 413 | ANXA7_HUMAN | 1213 | H14_HUMAN | 2013 | PED1B_HUMAN |
| 414 | ANR17_HUMAN | 1214 | GNPI1_HUMAN | 2014 | PRSR2_HUMAN |
| 415 | AOAH_HUMAN | 1215 | GUC2C_HUMAN | 2015 | POK6_HUMAN |
| 416 | AP5B1_HUMAN | 1216 | GPR3_HUMAN | 2016 | PRRX1_HUMAN |
| 417 | APOA2_HUMAN | 1217 | GPSM1_HUMAN | 2017 | PSG5_HUMAN |
| 418 | ADA33_HUMAN | 1218 | P2RX2_HUMAN | 2018 | PTBP1_HUMAN |
| 419 | ADDA_HUMAN | 1219 | GPAM1_HUMAN | 2019 | VPS4A_HUMAN |
| 420 | ADT2_HUMAN | 1220 | RD21L_HUMAN | 2020 | PRA19_HUMAN |
| 421 | AGRE1_HUMAN | 1221 | GRAPL_HUMAN | 2021 | PRP8_HUMAN |
| 422 | AGRG5_HUMAN | 1222 | GSTP1_HUMAN | 2022 | PRR13_HUMAN |
| 423 | ALKB3_HUMAN | 1223 | HMCS2_HUMAN | 2023 | PRR11_HUMAN |
| 424 | ALS_HUMAN | 1224 | HME2_HUMAN | 2024 | PPP6_HUMAN |
| 425 | ZN439_HUMAN | 1225 | HORM2_HUMAN | 2025 | PR20E_HUMAN |
| 426 | ADGB_HUMAN | 1226 | HOT_HUMAN | 2026 | PRCC_HUMAN |
| 427 | ADPRM_HUMAN | 1227 | HPIP_HUMAN | 2027 | PROX2_HUMAN |
| 428 | AJUBA_HUMAN | 1228 | HPT_HUMAN | 2028 | PS1C1_HUMAN |
| 429 | AKD1B_HUMAN | 1229 | HHATL_HUMAN | 2029 | S4A8_HUMAN |
| 430 | ACSF3_HUMAN | 1230 | HHLA2_HUMAN | 2030 | PP16B_HUMAN |
| 431 | AGRF3_HUMAN | 1231 | HMGB4_HUMAN | 2031 | QRIC1_HUMAN |
| 432 | ARSJ_HUMAN | 1232 | GLCTK_HUMAN | 2032 | PROB1_HUMAN |
| 433 | ASB6_HUMAN | 1233 | FKBP5_HUMAN | 2033 | PROL4_HUMAN |
| 434 | AN34B_HUMAN | 1234 | HNF4A_HUMAN | 2034 | PRP16_HUMAN |
| 435 | ARFG3_HUMAN | 1235 | HNRPL_HUMAN | 2035 | PPP5_HUMAN |
| 436 | ASB10_HUMAN | 1236 | HLPDA_HUMAN | 2036 | PRS35_HUMAN |
| 437 | ASB15_HUMAN | 1237 | GLT10_HUMAN | 2037 | RABL3_HUMAN |
| 438 | ZN155_HUMAN | 1238 | HDX_HUMAN | 2038 | PXDC1_HUMAN |
| 439 | ARCH_HUMAN | 1239 | TNR6_HUMAN | 2039 | RASF8_HUMAN |
| 440 | ARMC6_HUMAN | 1240 | TOPRS_HUMAN | 2040 | RBM34_HUMAN |
| 441 | ARL2_HUMAN | 1241 | GMCL1_HUMAN | 2041 | RBNS5_HUMAN |
| 442 | ARMC1_HUMAN | 1242 | GLPE_HUMAN | 2042 | QSOX1_HUMAN |
| 443 | ARMC3_HUMAN | 1243 | H32_HUMAN | 2043 | R113B_HUMAN |
| 444 | ARPIN_HUMAN | 1244 | H6ST3_HUMAN | 2044 | RAB2A_HUMAN |
| 445 | ASB3_HUMAN | 1245 | MLRA_HUMAN | 2045 | RALY_HUMAN |
| 446 | AMPN_HUMAN | 1246 | HDAC6_HUMAN | 2046 | CCD15_HUMAN |
| 447 | ANKF1_HUMAN | 1247 | HEAT9_HUMAN | 2047 | RB40A_HUMAN |
| 448 | LEU7_HUMAN | 1248 | HEP2_HUMAN | 2048 | QSPP_HUMAN |
| 449 | SPC25_HUMAN | 1249 | IP3KA_HUMAN | 2049 | RABP1_HUMAN |
| 450 | AP1AR_HUMAN | 1250 | H2B2C_HUMAN | 2050 | PTN3_HUMAN |
| 451 | FA71D_HUMAN | 1251 | HECW2_HUMAN | 2051 | RBGP1_HUMAN |
| 452 | ANR33_HUMAN | 1252 | HMX2_HUMAN | 2052 | RDH1_HUMAN |
| 453 | ASTN1_HUMAN | 1253 | HMN3_HUMAN | 2053 | RAD9B_HUMAN |
| 454 | AL3B2_HUMAN | 1254 | LRC55_HUMAN | 2054 | REC9_HUMAN |
| 455 | RBBP4_HUMAN | 1255 | H2B1C_HUMAN | 2055 | RBY1E_HUMAN |
| 456 | ANF_HUMAN | 1256 | HPPD_HUMAN | 2056 | SC61B_HUMAN |
| 457 | ZDHC5_HUMAN | 1257 | MIF_HUMAN | 2057 | RAP1A_HUMAN |
| 458 | AN32A_HUMAN | 1258 | MDN1_HUMAN | 2058 | RBM44_HUMAN |
| 459 | KPCB_HUMAN | 1259 | MFAP1_HUMAN | 2059 | TT39B_HUMAN |
| 460 | POTB2_HUMAN | 1260 | MEF2C_HUMAN | 2060 | RAB6A_HUMAN |
| 461 | ATL2_HUMAN | 1261 | MK07_HUMAN | 2061 | RCCD1_HUMAN |
| 462 | ATPUN_HUMAN | 1262 | MTR1A_HUMAN | 2062 | REG1B_HUMAN |
| 463 | AT5G2_HUMAN | 1263 | MORN4_HUMAN | 2063 | RBM24_HUMAN |
| 464 | AT8B1_HUMAN | 1264 | MPPA_HUMAN | 2064 | RBM8A_HUMAN |
| 465 | ATS18_HUMAN | 1265 | MPRIP_HUMAN | 2065 | RBP1_HUMAN |
| 466 | ATS6_HUMAN | 1266 | MRCKB_HUMAN | 2066 | RENI_HUMAN |
| 467 | ATS20_HUMAN | 1267 | HNF6_HUMAN | 2067 | RASF6_HUMAN |
| 468 | NCEH1_HUMAN | 1268 | HNRPM_HUMAN | 2068 | RBMS2_HUMAN |
| 469 | ARP5_HUMAN | 1269 | I23O2_HUMAN | 2069 | RBSK_HUMAN |
| 470 | AT1A3_HUMAN | 1270 | ICA69_HUMAN | 2070 | RD3_HUMAN |
| 471 | AT2B3_HUMAN | 1271 | HXA3_HUMAN | 2071 | RFA1_HUMAN |
| 472 | ATP23_HUMAN | 1272 | HXB6_HUMAN | 2072 | RASD1_HUMAN |
| 473 | MIER1_HUMAN | 1273 | HYI_HUMAN | 2073 | S17A4_HUMAN |
| 474 | IFN10_HUMAN | 1274 | IBP2_HUMAN | 2074 | RHG15_HUMAN |
| 475 | ARRD2_HUMAN | 1275 | VP9D1_HUMAN | 2075 | RHG10_HUMAN |
| 476 | ARRS_HUMAN | 1276 | DUS26_HUMAN | 2076 | RHOD_HUMAN |
| 477 | ADCY1_HUMAN | 1277 | HNRC1_HUMAN | 2077 | RINL_HUMAN |
| 478 | AGAP5_HUMAN | 1278 | HGNAT_HUMAN | 2078 | RET1_HUMAN |
| 479 | AGTR1_HUMAN | 1279 | HUS1B_HUMAN | 2079 | RFIP4_HUMAN |
| 480 | ASB11_HUMAN | 1280 | IFN21_HUMAN | 2080 | RFNG_HUMAN |
| 481 | ARL17_HUMAN | 1281 | IFT57_HUMAN | 2081 | RFPLA_HUMAN |
| 482 | ARL1_HUMAN | 1282 | IGHG1_HUMAN | 2082 | RFTN2_HUMAN |
| 483 | ASGL1_HUMAN | 1283 | IGHM_HUMAN | 2083 | RGDSR_HUMAN |
| 484 | MRAP2_HUMAN | 1284 | HS902_HUMAN | 2084 | RL12_HUMAN |
| 485 | ATF3_HUMAN | 1285 | HSPB9_HUMAN | 2085 | REL2_HUMAN |
| 486 | TBC3I_HUMAN | 1286 | HV311_HUMAN | 2086 | RPC7L_HUMAN |
| 487 | CASQ1_HUMAN | 1287 | HV343_HUMAN | 2087 | RN175_HUMAN |
| 488 | AT2L2_HUMAN | 1288 | HV692_HUMAN | 2088 | VITRN_HUMAN |
| 489 | AT5F1_HUMAN | 1289 | IFIH1_HUMAN | 2089 | RPC6_HUMAN |
| 490 | ARRD1_HUMAN | 1290 | IFT43_HUMAN | 2090 | RPP40_HUMAN |
| 491 | ASA2B_HUMAN | 1291 | ISK9_HUMAN | 2091 | RPRML_HUMAN |
| 492 | AT2B2_HUMAN | 1292 | INT6_HUMAN | 2092 | RERE_HUMAN |
| 493 | ATOH8_HUMAN | 1293 | IQCC_HUMAN | 2093 | PTF1A_HUMAN |
| 494 | ATPA_HUMAN | 1294 | I17RB_HUMAN | 2094 | PMS2L_HUMAN |
| 495 | ATRIP_HUMAN | 1295 | ICOSL_HUMAN | 2095 | PTPRC_HUMAN |
| 496 | BCL9_HUMAN | 1296 | ID4_HUMAN | 2096 | RGS7_HUMAN |
| 497 | BAAS2_HUMAN | 1297 | IFT81_HUMAN | 2097 | PPOX_HUMAN |
| 498 | BAG1_HUMAN | 1298 | IGFL3_HUMAN | 2098 | PPR21_HUMAN |
| 499 | BEND3_HUMAN | 1299 | INT1_HUMAN | 2099 | RHBL2_HUMAN |
| 500 | BARD1_HUMAN | 1300 | IFNK_HUMAN | 2100 | PR20D_HUMAN |
| 501 | BHMT1_HUMAN | 1301 | IGS21_HUMAN | 2101 | RFX5_HUMAN |
| 502 | BIEA_HUMAN | 1302 | IL17C_HUMAN | 2102 | FKBP3_HUMAN |
| 503 | BIG1_HUMAN | 1303 | OR5DG_HUMAN | 2103 | RGS19_HUMAN |
| 504 | AXA2L_HUMAN | 1304 | MMP20_HUMAN | 2104 | PREY_HUMAN |
| 505 | BASI_HUMAN | 1305 | IL7_HUMAN | 2105 | RIPK4_HUMAN |
| 506 | BBS1_HUMAN | 1306 | IMP3_HUMAN | 2106 | RL1D1_HUMAN |
| 507 | BAGE2_HUMAN | 1307 | I17RA_HUMAN | 2107 | RELN_HUMAN |
| 508 | BANP_HUMAN | 1308 | IL31R_HUMAN | 2108 | RL15_HUMAN |
| 509 | BAZ2B_HUMAN | 1309 | IL33_HUMAN | 2109 | RL31_HUMAN |
| 510 | BRE1A_HUMAN | 1310 | IGSF1_HUMAN | 2110 | RPB3_HUMAN |
| 511 | BRF2_HUMAN | 1311 | IKKA_HUMAN | 2111 | RORA_HUMAN |
| 512 | BTNL2_HUMAN | 1312 | IDLC_HUMAN | 2112 | RPB11_HUMAN |
| 513 | BUB3_HUMAN | 1313 | IF172_HUMAN | 2113 | GRHPR_HUMAN |
| 514 | C102B_HUMAN | 1314 | IF2B3_HUMAN | 2114 | AMOL2_HUMAN |
| 515 | BAT1_HUMAN | 1315 | IF5A1_HUMAN | 2115 | RPP30_HUMAN |
| 516 | BATF3_HUMAN | 1316 | IL18_HUMAN | 2116 | HES1_HUMAN |
| 517 | BICD1_HUMAN | 1317 | IMB1_HUMAN | 2117 | RS15A_HUMAN |
| 518 | BMF_HUMAN | 1318 | METK1_HUMAN | 2118 | RMD2_HUMAN |
| 519 | BLACE_HUMAN | 1319 | MFRN1_HUMAN | 2119 | RN121_HUMAN |
| 520 | BMP15_HUMAN | 1320 | MFSD1_HUMAN | 2120 | RN138_HUMAN |
| 521 | BMPER_HUMAN | 1321 | MI1HG_HUMAN | 2121 | RNS13_HUMAN |
| 522 | BMX_HUMAN | 1322 | MIC10_HUMAN | 2122 | RN170_HUMAN |
| 523 | ESPB1_HUMAN | 1323 | MMP3_HUMAN | 2123 | RPC7_HUMAN |
| 524 | BEND4_HUMAN | 1324 | MLH3_HUMAN | 2124 | RRP15_HUMAN |
| 525 | BGLR_HUMAN | 1325 | MMP14_HUMAN | 2125 | RS11_HUMAN |
| 526 | BLNK_HUMAN | 1326 | MMP25_HUMAN | 2126 | RCN1_HUMAN |
| 527 | BRAP_HUMAN | 1327 | MOC2A_HUMAN | 2127 | RUVB2_HUMAN |
| 528 | C11B1_HUMAN | 1328 | MOG_HUMAN | 2128 | S14L1_HUMAN |
| 529 | AURKB_HUMAN | 1329 | MIRO1_HUMAN | 2129 | RPIA_HUMAN |
| 530 | BFAR_HUMAN | 1330 | MKRN2_HUMAN | 2130 | RPTN_HUMAN |
| 531 | BEND5_HUMAN | 1331 | MLIP_HUMAN | 2131 | RRN3_HUMAN |
| 532 | BIK_HUMAN | 1332 | MORN2_HUMAN | 2132 | RPP29_HUMAN |
| 533 | ARF6_HUMAN | 1333 | MLP3A_HUMAN | 2133 | RS9_HUMAN |
| 534 | AZIN2_HUMAN | 1334 | MMP8_HUMAN | 2134 | RTEL1_HUMAN |
| 535 | BACD2_HUMAN | 1335 | MP2K4_HUMAN | 2135 | RTL9_HUMAN |
| 536 | BAIP3_HUMAN | 1336 | MP2K6_HUMAN | 2136 | RSLAB_HUMAN |
| 537 | AUNIP_HUMAN | 1337 | ML12B_HUMAN | 2137 | RSLBB_HUMAN |
| 538 | C1QR1_HUMAN | 1338 | MSS51_HUMAN | 2138 | RUNX2_HUMAN |
| 539 | C1T9B_HUMAN | 1339 | MLH1_HUMAN | 2139 | UBP51_HUMAN |
| 540 | BFSP1_HUMAN | 1340 | MLXIP_HUMAN | 2140 | RAD54_HUMAN |
| 541 | BLVRB_HUMAN | 1341 | MO2R2_HUMAN | 2141 | RALB_HUMAN |
| 542 | BRNP1_HUMAN | 1342 | MOAP1_HUMAN | 2142 | S31A3_HUMAN |
| 543 | BRX1_HUMAN | 1343 | MORC1_HUMAN | 2143 | S35B1_HUMAN |
| 544 | BHA09_HUMAN | 1344 | MIRH1_HUMAN | 2144 | RT10_HUMAN |
| 545 | CAN1_HUMAN | 1345 | MPCP_HUMAN | 2145 | RYBP_HUMAN |
| 546 | CAN2_HUMAN | 1346 | MPP3_HUMAN | 2146 | RT14_HUMAN |
| 547 | RBP2_HUMAN | 1347 | MGRN1_HUMAN | 2147 | S35B2_HUMAN |
| 548 | RBX2_HUMAN | 1348 | MKNK2_HUMAN | 2148 | S35B3_HUMAN |
| 549 | CBX8_HUMAN | 1349 | ML12A_HUMAN | 2149 | RHG31_HUMAN |
| 550 | CA146_HUMAN | 1350 | NXF2_HUMAN | 2150 | RHG32_HUMAN |
| 551 | CAPR2_HUMAN | 1351 | OARD1_HUMAN | 2151 | SATL1_HUMAN |
| 552 | CATB_HUMAN | 1352 | OGFR_HUMAN | 2152 | S23A2_HUMAN |
| 553 | C2C4C_HUMAN | 1353 | OPSD_HUMAN | 2153 | S2534_HUMAN |
| 554 | APBB3_HUMAN | 1354 | OR4Q2_HUMAN | 2154 | RX_HUMAN |
| 555 | APOL3_HUMAN | 1355 | OR7AH_HUMAN | 2155 | RTP3_HUMAN |
| 556 | CA053_HUMAN | 1356 | TSN15_HUMAN | 2156 | SCNNB_HUMAN |
| 557 | CABP4_HUMAN | 1357 | NXPH1_HUMAN | 2157 | RT28_HUMAN |
| 558 | INAM2_HUMAN | 1358 | OCAD2_HUMAN | 2158 | S15A1_HUMAN |
| 559 | BTAF1_HUMAN | 1359 | OR2M2_HUMAN | 2159 | S15A5_HUMAN |
| 560 | ASCC1_HUMAN | 1360 | OR2Y1_HUMAN | 2160 | RGPA2_HUMAN |
| 561 | BTG1_HUMAN | 1361 | OR3A1_HUMAN | 2161 | S22AD_HUMAN |
| 562 | CALM3_HUMAN | 1362 | AFF2_HUMAN | 2162 | S23IP_HUMAN |
| 563 | CAAP1_HUMAN | 1363 | AFTIN_HUMAN | 2163 | S29A4_HUMAN |
| 564 | CAPS1_HUMAN | 1364 | OR5F1_HUMAN | 2164 | SC22C_HUMAN |
| 565 | CAR17_HUMAN | 1365 | OR5K3_HUMAN | 2165 | S19A2_HUMAN |
| 566 | FIGL2_HUMAN | 1366 | ORC6_HUMAN | 2166 | AG10A_HUMAN |
| 567 | CA158_HUMAN | 1367 | OTU7A_HUMAN | 2167 | S1A7A_HUMAN |
| 568 | CA185_HUMAN | 1368 | NUTM1_HUMAN | 2168 | S35D2_HUMAN |
| 569 | CABP8_HUMAN | 1369 | NXPH4_HUMAN | 2169 | SC6A3_HUMAN |
| 570 | BCORL_HUMAN | 1370 | NXT2_HUMAN | 2170 | S12A4_HUMAN |
| 571 | ATG10_HUMAN | 1371 | O10J3_HUMAN | 2171 | S26A6_HUMAN |
| 572 | BCAT1_HUMAN | 1372 | O13C9_HUMAN | 2172 | SCN1A_HUMAN |
| 573 | BCAT2_HUMAN | 1373 | O52M1_HUMAN | 2173 | SCRN2_HUMAN |
| 574 | BWR1B_HUMAN | 1374 | ODFP2_HUMAN | 2174 | RTL3_HUMAN |
| 575 | ARFP2_HUMAN | 1375 | OR1P1_HUMAN | 2175 | RUSC1_HUMAN |
| 576 | CAC1C_HUMAN | 1376 | MMS19_HUMAN | 2176 | S39A6_HUMAN |
| 577 | BOLA1_HUMAN | 1377 | OR4P4_HUMAN | 2177 | S10A1_HUMAN |
| 578 | BORC6_HUMAN | 1378 | OR5M1_HUMAN | 2178 | ZCCHL_HUMAN |
| 579 | BORG2_HUMAN | 1379 | OR7D2_HUMAN | 2179 | S23A3_HUMAN |
| 580 | BORG5_HUMAN | 1380 | OR8D1_HUMAN | 2180 | S35A3_HUMAN |
| 581 | BSND_HUMAN | 1381 | OR2B8_HUMAN | 2181 | S35E2_HUMAN |
| 582 | BSPH1_HUMAN | 1382 | OR2J2_HUMAN | 2182 | S100G_HUMAN |
| 583 | C295L_HUMAN | 1383 | NUPL2_HUMAN | 2183 | S39A5_HUMAN |
| 584 | CA216_HUMAN | 1384 | O10A7_HUMAN | 2184 | S7A13_HUMAN |
| 585 | AGO3_HUMAN | 1385 | O10J5_HUMAN | 2185 | SAA1_HUMAN |
| 586 | CAH6_HUMAN | 1386 | MOB1A_HUMAN | 2186 | SC24D_HUMAN |
| 587 | BRAS2_HUMAN | 1387 | O13C4_HUMAN | 2187 | SC6A5_HUMAN |
| 588 | BRCA2_HUMAN | 1388 | O2AP1_HUMAN | 2188 | SEM4B_HUMAN |
| 589 | BRPF3_HUMAN | 1389 | OGRL1_HUMAN | 2189 | PRS42_HUMAN |
| 590 | C144A_HUMAN | 1390 | OSBP1_HUMAN | 2190 | RBM11_HUMAN |
| 591 | C1AS1_HUMAN | 1391 | OR5C1_HUMAN | 2191 | SEM6C_HUMAN |
| 592 | C5AR1_HUMAN | 1392 | PIGP_HUMAN | 2192 | SC5A1_HUMAN |
| 593 | CA127_HUMAN | 1393 | RAB23_HUMAN | 2193 | SCG2_HUMAN |
| 594 | CA196_HUMAN | 1394 | PACRL_HUMAN | 2194 | SCN2B_HUMAN |
| 595 | CABP2_HUMAN | 1395 | P4R3A_HUMAN | 2195 | RGMB_HUMAN |
| 596 | CABP5_HUMAN | 1396 | PAGE3_HUMAN | 2196 | SIM12_HUMAN |
| 597 | CC030_HUMAN | 1397 | MID49_HUMAN | 2197 | MBOA4_HUMAN |
| 598 | CC080_HUMAN | 1398 | PALM2_HUMAN | 2198 | SEM4C_HUMAN |
| 599 | CC103_HUMAN | 1399 | PA2G5_HUMAN | 2199 | SEPT8_HUMAN |
| 600 | CC130_HUMAN | 1400 | OGFD1_HUMAN | 2200 | SGTB_HUMAN |
| 601 | CC162_HUMAN | 1401 | PCDB8_HUMAN | 2201 | SMCA2_HUMAN |
| 602 | CCD69_HUMAN | 1402 | PAXX_HUMAN | 2202 | S47A2_HUMAN |
| 603 | CCL17_HUMAN | 1403 | PCDBG_HUMAN | 2203 | SIA8C_HUMAN |
| 604 | CCL19_HUMAN | 1404 | PDZD7_HUMAN | 2204 | S6A19_HUMAN |
| 605 | CCL25_HUMAN | 1405 | PE2R2_HUMAN | 2205 | S6A20_HUMAN |
| 606 | CCL5_HUMAN | 1406 | PDIA6_HUMAN | 2206 | SLAF9_HUMAN |
| 607 | CD79A_HUMAN | 1407 | PE2R4_HUMAN | 2207 | SIAT2_HUMAN |
| 608 | CD9_HUMAN | 1408 | PGFS_HUMAN | 2208 | SIX3_HUMAN |
| 609 | CDAN1_HUMAN | 1409 | PTHY_HUMAN | 2209 | SDF1_HUMAN |
| 610 | CDC45_HUMAN | 1410 | PGBD1_HUMAN | 2210 | SDS3_HUMAN |
| 611 | CEA19_HUMAN | 1411 | PTN14_HUMAN | 2211 | SG11A_HUMAN |
| 612 | CENPJ_HUMAN | 1412 | RAB17_HUMAN | 2212 | SCFD2_HUMAN |
| 613 | CERS4_HUMAN | 1413 | PYGM_HUMAN | 2213 | SDE2_HUMAN |
| 614 | CACO2_HUMAN | 1414 | PZRN4_HUMAN | 2214 | SESN1_HUMAN |
| 615 | CE126_HUMAN | 1415 | RALA_HUMAN | 2215 | IL17_HUMAN |
| 616 | DUTL_HUMAN | 1416 | PSD4_HUMAN | 2216 | SIAT6_HUMAN |
| 617 | CETP_HUMAN | 1417 | PSN1_HUMAN | 2217 | SC5D_HUMAN |
| 618 | CF120_HUMAN | 1418 | PTPRH_HUMAN | 2218 | SCAM3_HUMAN |
| 619 | CF222_HUMAN | 1419 | RHOV_HUMAN | 2219 | SC2B2_HUMAN |
| 620 | CFA46_HUMAN | 1420 | PSF2_HUMAN | 2220 | LVX54_HUMAN |
| 621 | CG072_HUMAN | 1421 | PSMD7_HUMAN | 2221 | SEH1_HUMAN |
| 622 | DVL1_HUMAN | 1422 | PSPN_HUMAN | 2222 | LCE5A_HUMAN |
| 623 | DYH10_HUMAN | 1423 | PT100_HUMAN | 2223 | LCN9_HUMAN |
| 624 | MEIOB_HUMAN | 1424 | PTGR1_HUMAN | 2224 | B3GN5_HUMAN |
| 625 | CHIP_HUMAN | 1425 | PTH1R_HUMAN | 2225 | SFT2C_HUMAN |
| 626 | CAR18_HUMAN | 1426 | PTPRN_HUMAN | 2226 | RYDEN_HUMAN |
| 627 | CC169_HUMAN | 1427 | PTRD1_HUMAN | 2227 | SH321_HUMAN |
| 628 | CC173_HUMAN | 1428 | RNS12_HUMAN | 2228 | SH3G1_HUMAN |
| 629 | CD226_HUMAN | 1429 | RNZ1_HUMAN | 2229 | SIX6_HUMAN |
| 630 | CD27_HUMAN | 1430 | ROMO1_HUMAN | 2230 | SLIB_HUMAN |
| 631 | CELA1_HUMAN | 1431 | RP1_HUMAN | 2231 | SEPT9_HUMAN |
| 632 | MLRS_HUMAN | 1432 | RPAB2_HUMAN | 2232 | SGMR2_HUMAN |
| 633 | CDN2B_HUMAN | 1433 | ROBO1_HUMAN | 2233 | PKHB2_HUMAN |
| 634 | CE066_HUMAN | 1434 | RPA1_HUMAN | 2234 | SH2D6_HUMAN |
| 635 | CE104_HUMAN | 1435 | IPYR2_HUMAN | 2235 | SH3R3_HUMAN |
| 636 | CE170_HUMAN | 1436 | IQCAL_HUMAN | 2236 | SIM16_HUMAN |
| 637 | CAR16_HUMAN | 1437 | ROGDI_HUMAN | 2237 | S10A4_HUMAN |
| 638 | CARD8_HUMAN | 1438 | ROR2_HUMAN | 2238 | S4A4_HUMAN |
| 639 | CARM1_HUMAN | 1439 | RM43_HUMAN | 2239 | S52A3_HUMAN |
| 640 | CATD_HUMAN | 1440 | RL7A_HUMAN | 2240 | SAV1_HUMAN |
| 641 | CDD_HUMAN | 1441 | ISX_HUMAN | 2241 | SCN3B_HUMAN |
| 642 | CG066_HUMAN | 1442 | JPH2_HUMAN | 2242 | SNX20_HUMAN |
| 643 | C3P1_HUMAN | 1443 | K1C17_HUMAN | 2243 | SON_HUMAN |
| 644 | THAP2_HUMAN | 1444 | K2022_HUMAN | 2244 | SOX3_HUMAN |
| 645 | C163B_HUMAN | 1445 | MTF1_HUMAN | 2245 | SF3A3_HUMAN |
| 646 | CA054_HUMAN | 1446 | KCC2D_HUMAN | 2246 | SN12L_HUMAN |
| 647 | CA105_HUMAN | 1447 | KATL1_HUMAN | 2247 | SIK1_HUMAN |
| 648 | CAH4_HUMAN | 1448 | KAIN_HUMAN | 2248 | SOX10_HUMAN |
| 649 | CAH8_HUMAN | 1449 | KCNN2_HUMAN | 2249 | SL9A9_HUMAN |
| 650 | CC067_HUMAN | 1450 | KDF1_HUMAN | 2250 | SMAL1_HUMAN |
| 651 | CC036_HUMAN | 1451 | IGS11_HUMAN | 2251 | SMPX_HUMAN |
| 652 | CC14B_HUMAN | 1452 | K0907_HUMAN | 2252 | SP202_HUMAN |
| 653 | CBPB2_HUMAN | 1453 | OXDA_HUMAN | 2253 | SPC1L_HUMAN |
| 654 | CBPB1_HUMAN | 1454 | IMPG1_HUMAN | 2254 | SRG2B_HUMAN |
| 655 | CC014_HUMAN | 1455 | INSL4_HUMAN | 2255 | SLAF1_HUMAN |
| 656 | CE295_HUMAN | 1456 | INSR2_HUMAN | 2256 | SMIM4_HUMAN |
| 657 | CC150_HUMAN | 1457 | IPP2L_HUMAN | 2257 | SNR48_HUMAN |
| 658 | CC167_HUMAN | 1458 | ISOC2_HUMAN | 2258 | SNX13_HUMAN |
| 659 | CC182_HUMAN | 1459 | ITA4_HUMAN | 2259 | SODC_HUMAN |
| 660 | CC85C_HUMAN | 1460 | K0754_HUMAN | 2260 | SPCS3_HUMAN |
| 661 | CD4_HUMAN | 1461 | K1549_HUMAN | 2261 | SLN13_HUMAN |
| 662 | CE027_HUMAN | 1462 | CNKR1_HUMAN | 2262 | SLX1_HUMAN |
| 663 | CAZA1_HUMAN | 1463 | ITPR2_HUMAN | 2263 | SPD2B_HUMAN |
| 664 | CBPC4_HUMAN | 1464 | KANK3_HUMAN | 2264 | UBXN1_HUMAN |
| 665 | CC113_HUMAN | 1465 | KAT2B_HUMAN | 2265 | SPG7_HUMAN |
| 666 | CCNI2_HUMAN | 1466 | KCC1G_HUMAN | 2266 | SPKAP_HUMAN |
| 667 | CD026_HUMAN | 1467 | KDM4E_HUMAN | 2267 | O7E24_HUMAN |
| 668 | CD045_HUMAN | 1468 | IL15_HUMAN | 2268 | O11A1_HUMAN |
| 669 | CD52_HUMAN | 1469 | IL36B_HUMAN | 2269 | SH2B1_HUMAN |
| 670 | CDK9_HUMAN | 1470 | JHD2C_HUMAN | 2270 | SIM29_HUMAN |
| 671 | CDKL5_HUMAN | 1471 | KAD4_HUMAN | 2271 | SIKE1_HUMAN |
| 672 | CDRT1_HUMAN | 1472 | KRIT1_HUMAN | 2272 | SLAF5_HUMAN |
| 673 | CENPO_HUMAN | 1473 | INHBC_HUMAN | 2273 | SMBP2_HUMAN |
| 674 | CENPB_HUMAN | 1474 | INO1_HUMAN | 2274 | SMCR8_HUMAN |
| 675 | PTN13_HUMAN | 1475 | IRS1_HUMAN | 2275 | SMCA4_HUMAN |
| 676 | CE290_HUMAN | 1476 | IL8_HUMAN | 2276 | SMUG1_HUMAN |
| 677 | RAD52_HUMAN | 1477 | IRF7_HUMAN | 2277 | FKBPL_HUMAN |
| 678 | BORA_HUMAN | 1478 | KAD2_HUMAN | 2278 | SMCE1_HUMAN |
| 679 | CADH2_HUMAN | 1479 | NOD2_HUMAN | 2279 | SMDC1_HUMAN |
| 680 | DTX3L_HUMAN | 1480 | ITK_HUMAN | 2280 | SOSSC_HUMAN |
| 681 | CATA_HUMAN | 1481 | INKA2_HUMAN | 2281 | SOX12_HUMAN |
| 682 | CC056_HUMAN | 1482 | INMT_HUMAN | 2282 | SPE39_HUMAN |
| 683 | CC105_HUMAN | 1483 | IPP2_HUMAN | 2283 | SPHK2_HUMAN |
| 684 | CC189_HUMAN | 1484 | IQEC1_HUMAN | 2284 | SPP2A_HUMAN |
| 685 | CBPC2_HUMAN | 1485 | R51A2_HUMAN | 2285 | SHOX2_HUMAN |
| 686 | CBP_HUMAN | 1486 | INT7_HUMAN | 2286 | SIA7F_HUMAN |
| 687 | CCL4_HUMAN | 1487 | IPPK_HUMAN | 2287 | SIAH3_HUMAN |
| 688 | CD11B_HUMAN | 1488 | IRF1_HUMAN | 2288 | SLIK1_HUMAN |
| 689 | CD3E_HUMAN | 1489 | IRF6_HUMAN | 2289 | SPT2_HUMAN |
| 690 | CD3Z_HUMAN | 1490 | CR063_HUMAN | 2290 | SQOR_HUMAN |
| 691 | CD5R2_HUMAN | 1491 | KC1AL_HUMAN | 2291 | SRCA_HUMAN |
| 692 | CDK10_HUMAN | 1492 | KCC1D_HUMAN | 2292 | SRP72_HUMAN |
| 693 | CD166_HUMAN | 1493 | K1C14_HUMAN | 2293 | STMD1_HUMAN |
| 694 | CD248_HUMAN | 1494 | RAMP2_HUMAN | 2294 | T2R45_HUMAN |
| 695 | CCNL2_HUMAN | 1495 | KCNKI_HUMAN | 2295 | SGE2P_HUMAN |
| 696 | CCSE1_HUMAN | 1496 | IP6K2_HUMAN | 2296 | SPRE1_HUMAN |
| 697 | CD69_HUMAN | 1497 | IPO4_HUMAN | 2297 | SMAD3_HUMAN |
| 698 | CENPF_HUMAN | 1498 | ITA10_HUMAN | 2298 | SPT32_HUMAN |
| 699 | HERC3_HUMAN | 1499 | K0040_HUMAN | 2299 | SNPC3_HUMAN |
| 700 | MFAP3_HUMAN | 1500 | K1C23_HUMAN | 2300 | SO5A1_HUMAN |
| 701 | CERS1_HUMAN | 1501 | IRPL1_HUMAN | 2301 | SPG16_HUMAN |
| 702 | CI016_HUMAN | 1502 | K1C19_HUMAN | 2302 | SPI2A_HUMAN |
| 703 | CFA70_HUMAN | 1503 | KR131_HUMAN | 2303 | SPIC_HUMAN |
| 704 | CG077_HUMAN | 1504 | K1107_HUMAN | 2304 | SMCO2_HUMAN |
| 705 | CHD8_HUMAN | 1505 | KCNK2_HUMAN | 2305 | SRSF9_HUMAN |
| 706 | CHIT1_HUMAN | 1506 | PSG1_HUMAN | 2306 | SMAD4_HUMAN |
| 707 | CHSS1_HUMAN | 1507 | SETB1_HUMAN | 2307 | SUMF1_HUMAN |
| 708 | CI072_HUMAN | 1508 | ITAV_HUMAN | 2308 | SMAD9_HUMAN |
| 709 | CJ091_HUMAN | 1509 | KCNG2_HUMAN | 2309 | SPB6_HUMAN |
| 710 | CJ107_HUMAN | 1510 | KCNK1_HUMAN | 2310 | SPRC_HUMAN |
| 711 | CGB3_HUMAN | 1511 | KCNQ3_HUMAN | 2311 | STMN3_HUMAN |
| 712 | CHM4C_HUMAN | 1512 | KGP1_HUMAN | 2312 | RHAG_HUMAN |
| 713 | CHST1_HUMAN | 1513 | KI2LB_HUMAN | 2313 | SREC_HUMAN |
| 714 | CI129_HUMAN | 1514 | KI3S1_HUMAN | 2314 | SVOPL_HUMAN |
| 715 | CDCA3_HUMAN | 1515 | KCNH3_HUMAN | 2315 | SPOC1_HUMAN |
| 716 | CEP85_HUMAN | 1516 | KDM4C_HUMAN | 2316 | SPPL3_HUMAN |
| 717 | CF218_HUMAN | 1517 | KDM7A_HUMAN | 2317 | SPRE2_HUMAN |
| 718 | CO4A_HUMAN | 1518 | F107B_HUMAN | 2318 | SPS1_HUMAN |
| 719 | CEP68_HUMAN | 1519 | KLF9_HUMAN | 2319 | SRAC1_HUMAN |
| 720 | CHAD_HUMAN | 1520 | KXDL1_HUMAN | 2320 | SSFA2_HUMAN |
| 721 | CHKB_HUMAN | 1521 | KRBX1_HUMAN | 2321 | CLIC3_HUMAN |
| 722 | CING_HUMAN | 1522 | KS6A3_HUMAN | 2322 | SSPN_HUMAN |
| 723 | CIP2A_HUMAN | 1523 | KCNE3_HUMAN | 2323 | STX1A_HUMAN |
| 724 | CEL2A_HUMAN | 1524 | KCNT2_HUMAN | 2324 | STX4_HUMAN |
| 725 | CE056_HUMAN | 1525 | JERKY_HUMAN | 2325 | ST6B1_HUMAN |
| 726 | CENPX_HUMAN | 1526 | KIF3C_HUMAN | 2326 | TFF2_HUMAN |
| 727 | CN178_HUMAN | 1527 | KMT5C_HUMAN | 2327 | STIM1_HUMAN |
| 728 | CF157_HUMAN | 1528 | P121C_HUMAN | 2328 | STML2_HUMAN |
| 729 | CFC1_HUMAN | 1529 | K2C72_HUMAN | 2329 | STX17_HUMAN |
| 730 | CHST8_HUMAN | 1530 | CATS_HUMAN | 2330 | SUIS_HUMAN |
| 731 | CI057_HUMAN | 1531 | BTBD3_HUMAN | 2331 | SUMO1_HUMAN |
| 732 | CLC4G_HUMAN | 1532 | KR212_HUMAN | 2332 | SYCP1_HUMAN |
| 733 | CLD3_HUMAN | 1533 | BTD_HUMAN | 2333 | STK35_HUMAN |
| 734 | CO4A1_HUMAN | 1534 | BUD31_HUMAN | 2334 | STML3_HUMAN |
| 735 | CJ142_HUMAN | 1535 | MYLK_HUMAN | 2335 | SUV91_HUMAN |
| 736 | CK097_HUMAN | 1536 | MTCH1_HUMAN | 2336 | SYLM_HUMAN |
| 737 | CLOCK_HUMAN | 1537 | NAB1_HUMAN | 2337 | SSR5_HUMAN |
| 738 | CMAH_HUMAN | 1538 | NHLC1_HUMAN | 2338 | T126A_HUMAN |
| 739 | CEMP1_HUMAN | 1539 | KRA57_HUMAN | 2339 | ST1A4_HUMAN |
| 740 | SHOT1_HUMAN | 1540 | NADC_HUMAN | 2340 | ST3L2_HUMAN |
| 741 | CHD1L_HUMAN | 1541 | KLAS1_HUMAN | 2341 | STAC2_HUMAN |
| 742 | CHUR_HUMAN | 1542 | KLDC4_HUMAN | 2342 | SUCB1_HUMAN |
| 743 | CI084_HUMAN | 1543 | KLF12_HUMAN | 2343 | SYFB_HUMAN |
| 744 | CK001_HUMAN | 1544 | KLHL3_HUMAN | 2344 | SYJ2B_HUMAN |
| 745 | CL18B_HUMAN | 1545 | F170A_HUMAN | 2345 | STAM2_HUMAN |
| 746 | CL20A_HUMAN | 1546 | KBTBC_HUMAN | 2346 | SSBP4_HUMAN |
| 747 | CLD2_HUMAN | 1547 | KC1G2_HUMAN | 2347 | STAR9_HUMAN |
| 748 | CLD4_HUMAN | 1548 | KMT2D_HUMAN | 2348 | STIP1_HUMAN |
| 749 | CLU_HUMAN | 1549 | GG6L6_HUMAN | 2349 | STX10_HUMAN |
| 750 | CNR2_HUMAN | 1550 | GGA3_HUMAN | 2350 | SURF1_HUMAN |
| 751 | CO056_HUMAN | 1551 | KPCE_HUMAN | 2351 | SYK_HUMAN |
| 752 | COF2_HUMAN | 1552 | KPCL_HUMAN | 2352 | TA2R8_HUMAN |
| 753 | COQ7_HUMAN | 1553 | KR111_HUMAN | 2353 | SUCHY_HUMAN |
| 754 | COR1A_HUMAN | 1554 | KR192_HUMAN | 2354 | TADBP_HUMAN |
| 755 | CL073_HUMAN | 1555 | LASP1_HUMAN | 2355 | T200A_HUMAN |
| 756 | CL18A_HUMAN | 1556 | K2013_HUMAN | 2356 | T106C_HUMAN |
| 757 | CLD18_HUMAN | 1557 | KRT35_HUMAN | 2357 | T151A_HUMAN |
| 758 | CLIC2_HUMAN | 1558 | KHK_HUMAN | 2358 | SY14L_HUMAN |
| 759 | CLP1L_HUMAN | 1559 | TGM1_HUMAN | 2359 | T170A_HUMAN |
| 760 | CLPP_HUMAN | 1560 | TESK2_HUMAN | 2360 | TBB4B_HUMAN |
| 761 | COMD1_HUMAN | 1561 | TEX49_HUMAN | 2361 | TDRD5_HUMAN |
| 762 | CNTLN_HUMAN | 1562 | TES_HUMAN | 2362 | TGFB3_HUMAN |
| 763 | CNTN4_HUMAN | 1563 | KCMB2_HUMAN | 2363 | TGM3_HUMAN |
| 764 | CNTP2_HUMAN | 1564 | KDM1A_HUMAN | 2364 | T2FB_HUMAN |
| 765 | CLRN1_HUMAN | 1565 | KLK11_HUMAN | 2365 | TF2H3_HUMAN |
| 766 | CIR1_HUMAN | 1566 | KRA21_HUMAN | 2366 | T126B_HUMAN |
| 767 | CNCG_HUMAN | 1567 | KDM8_HUMAN | 2367 | T4S4_HUMAN |
| 768 | COQ6_HUMAN | 1568 | KLF14_HUMAN | 2368 | TAAR1_HUMAN |
| 769 | CODA1_HUMAN | 1569 | KLH24_HUMAN | 2369 | TAF11_HUMAN |
| 770 | SHAN2_HUMAN | 1570 | KLH35_HUMAN | 2370 | TBA1A_HUMAN |
| 771 | COL11_HUMAN | 1571 | KLHL2_HUMAN | 2371 | TBC30_HUMAN |
| 772 | COX20_HUMAN | 1572 | KLK15_HUMAN | 2372 | TALDO_HUMAN |
| 773 | COLL1_HUMAN | 1573 | KLK9_HUMAN | 2373 | TEAD1_HUMAN |
| 774 | CRBS_HUMAN | 1574 | KPSH2_HUMAN | 2374 | TELT_HUMAN |
| 775 | CLAP2_HUMAN | 1575 | KR105_HUMAN | 2375 | TCAL7_HUMAN |
| 776 | CLASR_HUMAN | 1576 | KR201_HUMAN | 2376 | TCF15_HUMAN |
| 777 | CLC1A_HUMAN | 1577 | THSD4_HUMAN | 2377 | TDR12_HUMAN |
| 778 | CLK1_HUMAN | 1578 | THOC6_HUMAN | 2378 | TEX2_HUMAN |
| 779 | CPVL_HUMAN | 1579 | FHL1_HUMAN | 2379 | TEX47_HUMAN |
| 780 | CN079_HUMAN | 1580 | FARP2_HUMAN | 2380 | TF2LX_HUMAN |
| 781 | CNBD2_HUMAN | 1581 | FANCB_HUMAN | 2381 | TA2R3_HUMAN |
| 782 | CNDP2_HUMAN | 1582 | L2HDH_HUMAN | 2382 | TBC12_HUMAN |
| 783 | CS082_HUMAN | 1583 | TOP2B_HUMAN | 2383 | TBC31_HUMAN |
| 784 | CO6_HUMAN | 1584 | TNPO3_HUMAN | 2384 | TBL1R_HUMAN |
| 785 | CO9_HUMAN | 1585 | KIRR3_HUMAN | 2385 | SYT14_HUMAN |
| 786 | CL097_HUMAN | 1586 | KRA13_HUMAN | 2386 | TECRL_HUMAN |
| 787 | F214B_HUMAN | 1587 | KRI1_HUMAN | 2387 | TAAR2_HUMAN |
| 788 | CX6A2_HUMAN | 1588 | LAP4A_HUMAN | 2388 | TBC23_HUMAN |
| 789 | COX15_HUMAN | 1589 | KRIP1_HUMAN | 2389 | TBC3E_HUMAN |
| 790 | CP2A7_HUMAN | 1590 | LIGO3_HUMAN | 2390 | YA026_HUMAN |
| 791 | COG5_HUMAN | 1591 | ECSIT_HUMAN | 2391 | YB003_HUMAN |
| 792 | COPA1_HUMAN | 1592 | KIF27_HUMAN | 2392 | YC018_HUMAN |
| 793 | COX2_HUMAN | 1593 | MFN2_HUMAN | 2393 | YF016_HUMAN |
| 794 | COX7C_HUMAN | 1594 | LIPB1_HUMAN | 2394 | YG055_HUMAN |
| 795 | CP3A7_HUMAN | 1595 | KCNV2_HUMAN | 2395 | YDJC_HUMAN |
| 796 | CP4AB_HUMAN | 1596 | FBXL5_HUMAN | 2396 | YAP1_HUMAN |
| 797 | CTGEF_HUMAN | 1597 | LMOD1_HUMAN | 2397 | YBOX2_HUMAN |
| 798 | COG1_HUMAN | 1598 | LN28A_HUMAN |  |  |
| 799 | ATIF1_HUMAN | 1599 | LAS1L_HUMAN |  |  |
| 800 | ATPF1_HUMAN | 1600 | KLF2_HUMAN |  |  |
|  |  |  |  |  |  |

| **Supplementary Table 3 35 genes enrichment in neurotrophin signaling pathway** | | | |
| --- | --- | --- | --- |
| **Gene** | **Cytogenetic Location** | **functional relevance to autism or ASD** | **Reference** |
| RPS6KA1 | 1p36.11 | CNV | Pinto, D., et al. (2010). "Functional impact of global rare copy number variation in autism spectrum disorders." Nature 466(7304): 368-372. |
| CDC42 | 1p36.12 | CNV | Bakos, J., et al. (2015). "Are molecules involved in neuritogenesis and axon guidance related to autism pathogenesis?" Neuromolecular medicine 17(3): 297-304. |
| TP73 | 1p36.3 | CNV | Gai, X., et al. (2012). "Rare structural variation of synapse and neurotransmission genes in autism." Mol Psychiatry 17(4): 402-411. |
| AKT3 | 1q43-q44 | CNV | Oliveira, B., et al. (2013). Synaptic transmission: looking for clues to autism spectrum disorders (ASD) etiology in copy number variants containing synaptic genes. European Human Genetics Conference, 8-11 june 2013, Instituto Nacional de Saúde Doutor Ricardo Jorge, IP. |
| PIK3CA | 3q26.32 | CNV | Auranen, M., et al. (2002). "A genomewide screen for autism-spectrum disorders: evidence for a major susceptibility locus on chromosome 3q25-27." Am J Hum Genet 71(4): 777-790. |
| GAB1 | 4q31.21 | CNV | Levy, D., et al. (2011). "Rare de novo and transmitted copy-number variation in autistic spectrum disorders." Neuron 70(5): 886-897. |
| MAPK14 | 6p21.31 | CNV | Anitha, A., et al. (2013). "Downregulation of the expression of mitochondrial electron transport complex genes in autism brains." Brain Pathology 23(3): 294-302. |
| RPS6KA2 | 6q27 | CNV | Berkel, S., et al. (2010). "Mutations in the SHANK2 synaptic scaffolding gene in autism spectrum disorder and mental retardation." Nat Genet 42(6): 489-491. |
| KRAS | 12p12.1 | CNV | Levy, D., et al. (2011). "Rare de novo and transmitted copy-number variation in autistic spectrum disorders." Neuron 70(5): 886-897. |
| IRAK4 | 12q12 | CNV | Wang, L. S., et al. (2010). "Population-based study of genetic variation in individuals with autism spectrum disorders from Croatia." BMC Med Genet 11 : 134. |
| PTPN11 | 12q24.13 | CNV | Betancur, C. (2011). "Etiological heterogeneity in autism spectrum disorders: more than 100 genetic and genomic disorders and still counting." Brain research 1380: 42-77. |
| SH2B1 | 16p11.2 | CNV | Tabet, A. C., et al. (2012). "Autism multiplex family with 16p11.2p12.2 microduplication syndrome in monozygotic twins and distal 16p11.2 deletion in their brother." Eur J Hum Genet 20(5): 540-546. |
| MAP3K3 | 17q23 | CNV | Levy, D., et al. (2011). "Rare de novo and transmitted copy-number variation in autistic spectrum disorders." Neuron 70(5): 886-897. |
| MAPK7 | 17p11.2 | CNV | Bremer, A., et al. (2011). "Copy number variation characteristics in subpopulations of patients with autism spectrum disorders." Am J Med Genet B Neuropsychiatr Genet 156(2): 115-124. |
| MAPK12 | 22q13.33 | CNV | Durand, C. M., et al. (2007). "Mutations in the gene encoding the synaptic scaffolding protein SHANK3 are associated with autism spectrum disorders." Nat Genet 39(1): 25-27. |
| PDPK1 | 16p13.3 | CNV | Ivon Cuscó., et al. (2009). Autism-specific copy number variants further implicate the phosphatidylinositol signaling pathway and the glutamatergic synapse in the etiology of the disorder.Hum Mol Genet. 2009 May 15;18(10):1795-804. |
| MAPK10 | 4q21.3 | Expression | Chow, M. L., et al. (2012). "Age-dependent brain gene expression and copy number anomalies in autism suggest distinct pathological processes at young versus mature ages." PLoS Genet 8(3): e1002592. |
| NFKB1 | 4q24 | Expression | Hu, V. W., et al. (2009). "Gene expression profiling differentiates autism case-controls and phenotypic variants of autism spectrum disorders: evidence for circadian rhythm dysfunction in severe autism." Autism Res 2(2): 78-97. |
| CAMK2D | 4q26 | Expression | Sullivan, J. M., et al. (2015). "Autism-like syndrome is induced by pharmacological suppression of BET proteins in young mice." J Exp Med 212(11): 1771-1781. |
| MAP3K1 | 5q11.2 | Expression | Zeidan-Chulia, F., et al. (2014). "Altered expression of Alzheimer's disease-related genes in the cerebellum of autistic patients: a model for disrupted brain connectome and therapy." Cell Death Dis 5: e1250. |
| ABL1 | 9q34.12 | Expression | Bakos, J., et al. (2015). "Are molecules involved in neuritogenesis and axon guidance related to autism pathogenesis?" Neuromolecular medicine 17(3): 297-304. |
| MAP2K5 | 15q23 | Expression | Shen, C., et al. (2011). "A proteomic investigation of B lymphocytes in an autistic family: a pilot study of exposure to natural rubber latex (NRL) may lead to autism." J Mol Neurosci 43(3): 443-452. |
| NTRK3 | 15q25.3 | Expression | Vardarajan, B. N., et al. (2013). "Haplotype structure enables prioritization of common markers and candidate genes in autism spectrum disorder." Transl Psychiatry 3: e262. |
| CRKL | 22q11.21 | Expression | Becker, E. and C. J. Stoodley (2013). "Autism spectrum disorder and the cerebellum." Int Rev Neurobiol 113: 1-34. |
| BCL2 | 18q21.33 | Methyaltion | Nguyen, A., et al. (2010). "Global methylation profiling of lymphoblastoid cell lines reveals epigenetic contributions to autism spectrum disorders and a novel autism candidate gene, RORA, whose protein product is reduced in autistic brain." Faseb j 24(8): 3036-3051. |
| MAPK1 | 22q11.22 | Mutation | Hormozdiari, F., et al. (2015). "The discovery of integrated gene networks for autism and related disorders." Genome research 25(1): 142-154. |
| CAMK2B | 7p13 | phosphorylation | Baucum, A. J., 2nd, et al. (2015). "Quantitative proteomics analysis of CaMKII phosphorylation and the CaMKII interactome in the mouse forebrain." ACS Chem Neurosci 6(4): 615-631. |
| PIK3R1 | 5q13.1 | Rare variants | Kelleher, R. J., 3rd, et al. (2012). "High-throughput sequencing of mGluR signaling pathway genes reveals enrichment of rare variants in autism." PLoS One 7(4): e35003. |
| IRS1 | 2q36.3 | SNP | Park, H. J., et al. (2016). "Association between IRS1 Gene polymorphism and autism Spectrum disorder: a pilot case-control study in Korean males." International journal of molecular sciences 17(8): 1227. |
| NTF3 | 12p13.31 | SNP | Toma, C., et al. (2013). "Neurotransmitter systems and neurotrophic factors in autism: association study of 37 genes suggests involvement of DDC." World J Biol Psychiatry 14(7): 516-527. |
| MAP2K7 | 19p13.2 |  |  |
| PLCG1 | 20q12 |  |  |
| PIK3R3 | 1p34.1 |  |  |
| SHC3 | 9q22.1 |  |  |
| BAD | 11q13.1 |  |  |

**Supplementary Table 4 Methylation status identified candidate sites of *SH2B1* in 30 pairs of sporadic ASD and matched controls by pyrosequencing**

| **Samples** | **Chr.16:28856729** | **Chr.16:28856735** | **Chr.16:28856743** | **Mean** |
| --- | --- | --- | --- | --- |
| **Autism** |  |  |  |  |
| **Control** |  |  |  |  |
| pair 1 | 0.6393 | 0.7797 | 0.7565 | 0.7252 |
|  | 0.6085 | 0.8093 | 0.7800 | 0.7326 |
| pair 2 | 0.5336 | 0.6891 | 0.6888 | 0.6372 |
|  | 0.5801 | 0.7168 | 0.7471 | 0.6813 |
| pair 3 | 0.5570 | 0.7150 | 0.7006 | 0.6575 |
|  | 0.5307 | 0.7018 | 0.7120 | 0.6482 |
| pair 4 | 0.5503 | 0.7267 | 0.7282 | 0.6684 |
|  | 0.6953 | 0.7543 | 0.8075 | 0.7524 |
| pair 5 | 0.5984 | 0.7309 | 0.7265 | 0.6853 |
|  | 0.5064 | 0.6876 | 0.5845 | 0.5928 |
| pair 6 | 0.9231 | 1.0000 | 1.0000 | 0.9744 |
|  | 0.6797 | 0.8093 | 0.7584 | 0.7491 |
| pair 7 | 0.6562 | 0.7761 | 0.8156 | 0.7493 |
|  | 0.7520 | 0.9221 | 0.9611 | 0.8784 |
| pair 8 | 0.6034 | 0.7668 | 0.7371 | 0.7024 |
|  | 0.6252 | 0.7900 | 0.7693 | 0.7282 |
| pair 9 | 0.5194 | 0.6950 | 0.7279 | 0.6474 |
|  | 0.4988 | 0.6746 | 0.6902 | 0.6212 |
| pair 10 | 0.8185 | 0.9166 | 0.9608 | 0.8986 |
|  | 0.4997 | 0.6473 | 0.6334 | 0.5935 |
| pair 11 | 0.6418 | 0.7988 | 0.8442 | 0.7616 |
|  | 0.6964 | 0.7738 | 0.8027 | 0.7576 |
| pair 12 | 0.6555 | 0.8233 | 0.7597 | 0.7462 |
|  | 0.5175 | 0.6737 | 0.6354 | 0.6089 |
| pair 13 | 0.6236 | 0.7662 | 0.7020 | 0.6973 |
|  | 0.5438 | 0.7115 | 0.6903 | 0.6485 |
| pair 14 | 0.6167 | 0.7838 | 0.7538 | 0.7181 |
|  | 0.6570 | 0.8490 | 0.8487 | 0.7849 |
| pair 15 | 0.5906 | 0.7389 | 0.7493 | 0.6929 |
|  | 0.5734 | 0.7165 | 0.7192 | 0.6697 |
| pair 16 | 0.6912 | 0.8097 | 0.8366 | 0.7792 |
|  | 0.5188 | 0.6675 | 0.6705 | 0.6189 |
| pair 17 | 0.5964 | 0.7846 | 0.6887 | 0.6899 |
|  | 0.6286 | 0.7715 | 0.7302 | 0.7101 |
| pair 18 | 0.6342 | 0.7999 | 0.8254 | 0.7532 |
|  | 0.7496 | 0.9340 | 0.9610 | 0.8815 |
| pair 19 | 0.8012 | 0.8838 | 0.9776 | 0.8875 |
|  | 0.4923 | 0.6588 | 0.6495 | 0.6002 |
| pair 20 | 0.6889 | 0.7957 | 0.7843 | 0.7563 |
|  | 0.4079 | 0.5975 | 0.5986 | 0.5347 |
| pair 21 | 0.6522 | 0.8230 | 0.7933 | 0.7562 |
|  | 0.6512 | 0.8029 | 0.7711 | 0.7417 |
| pair 22 | 0.7689 | 0.9012 | 0.9221 | 0.8641 |
|  | 0.7109 | 0.8304 | 0.7321 | 0.7578 |
| pair 23 | 0.5227 | 0.6417 | 0.6298 | 0.5981 |
|  | 0.5861 | 0.7353 | 0.7741 | 0.6985 |
| pair 24 | 0.6119 | 0.7729 | 0.8105 | 0.7318 |
|  | 0.5564 | 0.7216 | 0.7197 | 0.6659 |
| pair 25 | 0.7059 | 0.8275 | 0.8430 | 0.7921 |
|  | 0.5203 | 0.6831 | 0.6563 | 0.6199 |
| pair 26 | 0.5491 | 0.7065 | 0.7770 | 0.6775 |
|  | 0.6611 | 0.7986 | 0.6576 | 0.7058 |
| pair 27 | 0.5134 | 0.6606 | 0.6873 | 0.6204 |
|  | 0.5189 | 0.6637 | 0.6640 | 0.6155 |
| pair 28 | 0.7049 | 0.8706 | 0.9101 | 0.8285 |
|  | 0.4940 | 0.6246 | 0.6057 | 0.5748 |
| pair 29 | 0.6469 | 0.7856 | 0.7633 | 0.7319 |
|  | 0.7813 | 0.8682 | 0.9168 | 0.8554 |
| pair 30 | 0.6230 | 0.7714 | 0.8374 | 0.7439 |
|  | 0.6133 | 0.7842 | 0.7808 | 0.7261 |

**Supplementary Table 5 Cell-type composition adjustment in three ASD-discordant MZ twins**

| sample | CD8T | CD4T | NK | Bcell | Mono | Gran | *t*- test (p) |
| --- | --- | --- | --- | --- | --- | --- | --- |
| TP1_1 | 0.1390 | 0.1255 | 0.0000 | 0.0099 | 0.0710 | 0.5268 | 0.8667 |
| TP1_2 | 0.1808 | 0.0724 | 0.0409 | 0.0110 | 0.0729 | 0.5098 |  |
| TP2_1 | 0.1321 | 0.1263 | 0.0486 | 0.05817 | 0.0402 | 0.4903 | 0.9263 |
| TP2_2 | 0.1144 | 0.0833 | 0.0617 | 0.0319 | 0.0573 | 0.5564 |  |
| TP3_1 | 0.1699 | 0.0586 | 0.0364 | 0.0491 | 0.0392 | 0.5808 | 0.3832 |
| TP3_2 | 0.2028 | 0.0591 | 0.0474 | 0.0463 | 0.0268 | 0.5878 |  |

Note: TP: twin pair；_1:case, _2:control
